# Assessing countries’ social-ecological resilience to shifting marine commercial species

**DOI:** 10.1038/s41598-021-02328-6

**Published:** 2021-11-25

**Authors:** Elena Ojea, Elena Fontán, Isabel Fuentes-Santos, Juan Bueno-Pardo

**Affiliations:** 1 Centro de Investigación Mariña (CIM), Universidade de Vigo, Future Oceans Lab, Campus Lagoas Marcosende, 36310 Vigo, Spain; 2 Mareira Bizi Sociedade Cooperativa Galega, O Cruceiro, Briallos 23-B, 36658 Portas, Spain; 3 Consejo Superior de Investigaciones Científicas (CSIC), Instituto de Investigaciones Marinas (IIM), C/Eduardo Cabello, 6, 36208 Vigo, Spain

**Keywords:** Environmental impact, Climate-change adaptation, Climate-change policy

## Abstract

Climate change is already impacting fisheries with species moving across fishing areas, crossing institutional borders, and thus creating conflicts over fisheries management. In this scenario, scholars agree that adaptation to climate change requires that fisheries increase their social, institutional, and ecological resilience. The resilience or capacity of a fishery to be maintained without shifting to a different state (e.g., collapse) is at stake under climate change impacts and overexploitation. Despite this urgent need, applying the resilience concept in a spatially explicit and quantitative manner to inform policy remains unexplored. We take a resilience approach and operationalize the concept in industrial fisheries for two species that have been observed to significantly shift distribution in European waters: hake (*Merluccius merluccius*) and cod (*Gadus morhua*), in the context of the European Union institutional settings. With a set of resilience factors from the literature and by means of contemporary and historic data, we select indicators that are combined into an index that measures resilience on the ecologic, socioeconomic, and institutional dimensions of the fishery. We find that the resilience index varies among species and countries, with lower resilience levels in the socioeconomic dimension of the fisheries. We also see that resilience largely depends on the overexploitation status of the fishery. The results highlight the need to address social and institutional settings to enhance fisheries adaptation to climate change and allow to inform on climate resilient adaptation pathways for the fisheries.

## Introduction

Climate change causes very significant impacts on fisheries distribution and abundance around the world^1–6^, which are directly linked to ecological, socioeconomic and institutional consequences^7,8^. Evidence on climate change impacting European fish distribution is increasing, as suggested by the ascending number of scientific publications on this regard^9–13^. The International Council for the Exploration of the Sea (ICES) has released a special EU request report on shifts in stock distribution in European waters^14^, showing important shifts for some of the most valuable commercial species, including hake, cod, horse mackerel and anchovy. The EU aims to rebuild all fisheries to their Maximum Sustainable Yield (MSY) levels in order to meet the targets of both the Common Fisheries Policy and the Sustainable Development Goal 14.4, but neither of them were met by 2020. In this context, it is still unknown to what extent climate driven shifts in commercial species will continue to compromise this target. Meeting this goal will depend on the potential of EU countries to face the shifts in their targeted species, or in other words, on their resilience to shifting species. The present work contributes to answer this question with a resilience approach applied to EU fishing countries^15^.

In broad terms we understand resilience as the capacity of a fishery to cope with climate change impacts (e.g., a distributional shift) without changing to a different state (e.g., fisheries collapse). In climate change policy, vulnerability and risk assessments have been the main methodology used to inform decision making and adaptation^5,16–19^, as the vulnerability framework is adopted by the IPCC^20,21^. Vulnerability can be defined as the state of susceptibility to harm from climate change perturbations^17^. Vulnerability and risk assessments often reflect the state of a system and its components, such as fish species or fleets^19,22^. They usually do not consider the flexibility of the system to remain in the same state or to transform given external impacts^23,24^. This more dynamic view is in theory guaranteed with a resilience approach, but few examples exist on the fisheries literature that operationalize the resilience concept. Existing studies have looked at one single dimension (e.g., economic resilience^25^ or ecological resilience^26^) or focus on the ability of fishers to respond to changes (e.g.,^27,28^) or remain theoretical^29^. In this paper, we further develop resilience thinking to apply the concept by measuring the factors that have been identified in the literature to have an effect in fisheries resilience. We operationalize and integrate ecologic, socioeconomic, and institutional resilience, demonstrating that the approach can be applied from the stock level to the scale of fishing countries. This will allow to identify strengths and weaknesses in promoting resilience for different countries and marine species in the face of climate change and inform policy accordingly.

Indexes are very useful tools for conducting comparative analyses and raise awareness of an environmental problem^30^. Recent literature has developed quantitative applications of indexes for improving fisheries management^31^, social-ecological systems^32^ and climate change risk and vulnerability analyses^17^. However, none of these approaches considers the long-term trends in the fishery together with the capacity of the fishery to respond to climate change. In this line, recent work identifies a set of factors that can potentially increase resilience in a fishery^29,33,34^. These factors are obtained from a detailed analysis of the literature, where studies suggest and/or demonstrate a link between having those characteristics in a fishery and the resilience of that fishery to climate change. A comprehensive analysis on how these factors can build resilience and help identify adaptation pathways in a fishery has not yet been conducted, and the present work wants to address this gap.

## Resilience of fisheries

Resilience can be understood as the ability of a system to absorb disturbance without altering the fundamental structure, functions, and feedbacks of both its ecological and social components^35^. We understand ecological resilience as the ability of ecosystems or species to recover after a disturbance^29^, socioeconomic resilience as the capacity of a socioeconomic system to respond to the negative or adverse impacts and recover, as well as the capacity to adapt in case of stress or change^36^, and institutional resilience as the capacity of a natural resource governance system to absorb a disturbance while maintaining its major structures and functions^37^. We select two fisheries that have been significantly impacted by climate change in the EU, leading to shifts in their distribution in the last decades^14^. These species are European hake (*Merluccius merluccius*) and Atlantic cod (*Gadus morhua*). Recent literature on fisheries management adopts the resilience perspective and describes different settings and conditions within a fishery that enhance its ecological, social and/or institutional resilience^25,26,29,38^. We refer to these settings and conditions as ‘resilience factors’.

Table 1 lists the potential resilience factors identified from the literature that can measure the resilience of a fishery facing distributional shifts due to climate change. The ecological resilience of a stock may for example increase as the stock becomes more abundant, increasing spawning stock biomass (SSB), since more individuals can buffer some of the impacts of climate change^49^. In terms of socioeconomic resilience, a country that is very dependent on a specific stock that is vulnerable to climate change, may have lower resilience as compared to a country that has a diversified set of stocks harvested^50^. For institutional resilience, co-management normally implies more participation in decision-making and better capacity to adapt^41^. Despite this knowledge on potential resilience factors for a fishery, no previous studies have quantitatively operated resilience in this context. Having metrics about resilience can be crucial to fisheries management and climate change adaptation as it allows to compare different fisheries and countries to identify the factors that can be manipulated to enhance resilience^41^.

**Table 1:** List of resilience factors.

Factor name	Factor description	References	Effect on resilience
**Ecological Factors (increase resilience of the stock)**			
Area	Potential distribution area	^39,40^	The larger the distributional area and connectivity of a species the higher the buffer capacity to confront impacts
Abundance	Abundance trend	^41,42^	Stocks that have sustainable harvest over time and increase in abundance have greater capacity to adapt to a changing climate
Temperature	Temperature range of species	^1^	The larger the temperature range where the species can live the higher their flexibility and adaptability to changing ocean conditions
Age diversity	Age diverse target population	^29,39^	The truncation of age structure and loss of geographic substructure within populations makes stocks more sensitive to climate fluctuations
Overexploitation	Overexploitation	^43–45^	Fisheries that are overexploited are less resilient to climate change
Recovery	Recovery time	^26^	The larger the recovery time for a species the lower its resilience to adapt to climate change
**Socioeconomic factors (increase resilience of the fishery)**			
Gear diversity	Gear diversity	^44,45^	The number of different gear types that can be used in management increases flexibility and resilience
Fleet mobility	Fleet mobility	^25,33^	Distance that fleet can do to facilitate reaching the stocks increases the fishery resilience
Livelihood diversification	Livelihood diversification	^29,34^	Diverse sources of income allow fishers to assure their income under climate change
Fleet diversification	Fleet diversification (species)	^41^	The more species the fleet can catch the higher its resilience, as they can shift target species when impacted
Catch dependency	Catch dependency	^41^	The more dependent a country is on a particular stock in terms of landings, the less resilient to impacts in the fishery
Adaptive management	Adaptive management	^29,33,44^	The availability of scientific advice, management plans for sustainable exploitation or commercialization can help build resilience in a fishery as they promote sustainable harvest
**Institutional factors (increase resilience of the fishery and indirectly stocks)**			
Co-management	Institutions for fisheries co-management	^25,41,46^	The participation of fisheries and organizations with the public sector in managing the resource leads to more resilient fisheries
Property rights	Property rights (ITQs)	^29,38,47^	Having ownership and market flexibility over the stocks increases fishers and ecological resilience
Governance	Multi-level governance	^29,48^	Governance at different scales makes a flexible framework for adapting to change
Quotas	Catch quotas	^29,33,41^	Reinforces co-management if allocated together with other management tools in a context of management redundancy. Requires legislation and enforcement of legal frameworks, and cooperation of fisher-communities, which need to be adapted to countries and idiosyncrasies
Strength	Compliance and institutional strength	^24,41^	The degree of compliance with fisheries formal regulation rules in the country

Fisheries characteristics and management practices that have been seen to increase/decrease the resilience of a fishery to climate change.

Following previous applications of social-ecological theory^32^, we use indicators to measure the resilience factors. The indicators that we use to operationalize resilience are depicted in Table 2, while the methods, data sources and analysis are available in the SI and described in the methods section. All potential resilience factors from Table 1, except age diversity, livelihood and fleet diversification, and governance were introduced in the analysis, as data was accessible for both cod and hake fisheries at the required levels of analysis. We expect that fisheries resilience to shifting stock distribution has a spatial component where northern countries are more ecologically resilient as stocks are shifting northwards^14^. However, due to ocean institutions and borders in the EU, the social and institutional resilience could affect the ecological resilience, and the different dependencies of countries on the species can also play a role in their overall resilience index. We also expect the resilience index to have an opposite sign than that of fisheries vulnerability to climate change, as vulnerability and resilience theoretically have opposite directions, where vulnerability refers to the state of susceptibility to harm from perturbations^17^, and resilience refers to the ability of the system to absorb those perturbations.

**Table 2:** List of resilience indicators.

Factor	Indicator	Indicator description	DIR
E1. Area	E1.1 *Area2006*	Potential distribution area in all EEZs 2006 (km^2^)	+
	E1.2 *Area2100*	Potential distribution area in all EEZs 2100 (km^2^)	+
E2. Abundance	E2.1 *SSBhistoric*	Spawning Stock Biomass estimate of trend in SSB historic/SSB stock average	+
	E2.2 *SSBrecent*	Spawning Stock Biomass (SSB) trend in SSB 1980–2010/stock average SSB	+
	E2.3 *Ftrend*	Fishing Mortality (F) estimate of linear trend in historic F of stock/stock average F	−
	E2.4 *Rtrend*	Recruitment (R) estimate of linear trend in historic R of the stock/stock average R	+
E3. Temperature	E3.1 *T50*	Median preferred temperature (°C)	+
	E3.2 *Trange*	Range of preferred temperature (°C) (2nd and 98th percentiles)	+
E4. Overexploitation	E4.1 *OverMSY*	Index from B/Bmsy/F/Fmsy	−
	E4.2 *Status*	Position in F-Flimit/SSBSSBlimit plot (as in kobe plot)	+
E5. Recovery	E5.1 *Recovery*	Years since a stock biomass drops under SSBlimit and recovers back above this limit	−
S1. Gear diversity	S1.1 *SPgear*	Number of different gear types used by the fishery for the species	+
S2. Fleet mobility	S2.1 *ICESareas5*	Average number of ICES areas a fleet has accessed in the last 5 years	+
	S2.2 *ICESareasUE*	Difference in the number of ICES areas a fleet has accessed after and before of the EU started	+
S3. Catch dependency	S3.1 *Stockdep.sp*	Total catches of stock in country relative to total species catches in country	−
	S3.2 *Stockdep.total*	Total catches of stock in country divided by total catches in country	−
S4. Adaptive management	S4.1 *Research*	Investment in fisheries research in country	+
	S4.2 *Management*	Investment in fisheries management in country	+
I1. Co-management	I1.1 *N.organizations*	Number of producer organization in country	+
I2. Property rights	I2.1 *Swaps*	Money earned from quota exchanges in country	+
I3. Quotas	I3.2 *Above TAC*	Country catches above recommended TAC	−
I4. Strength	I4.2 *Compliance*	Inclusion of Requirements by country in 2010	+

The table shows the resilience factors from the literature and the proposed indicators for the analysis, with the direction (DIR) in which they influence resilience. See SI for greater details.

To measure fisheries resilience in commercial species we conduct a per stock and per country analysis of the resilience factors identified for the ecological, social, and institutional dimensions. The analysis at the stock level was only possible for the ecological dimension, as no data are available at the stock level for social and institutional indicators. EU fisheries management involves Total Allowable Catches (TAC) that manages a species over EU waters, where countries have quota allocations based on the stability principle^51^. Therefore, indicators such as the gears used, catch dependency, number of producer organizations, or the property rights are not associated to the specific stocks. We present the results per country for the socioeconomic and institutional dimensions.

## Results

**Figure 1:**
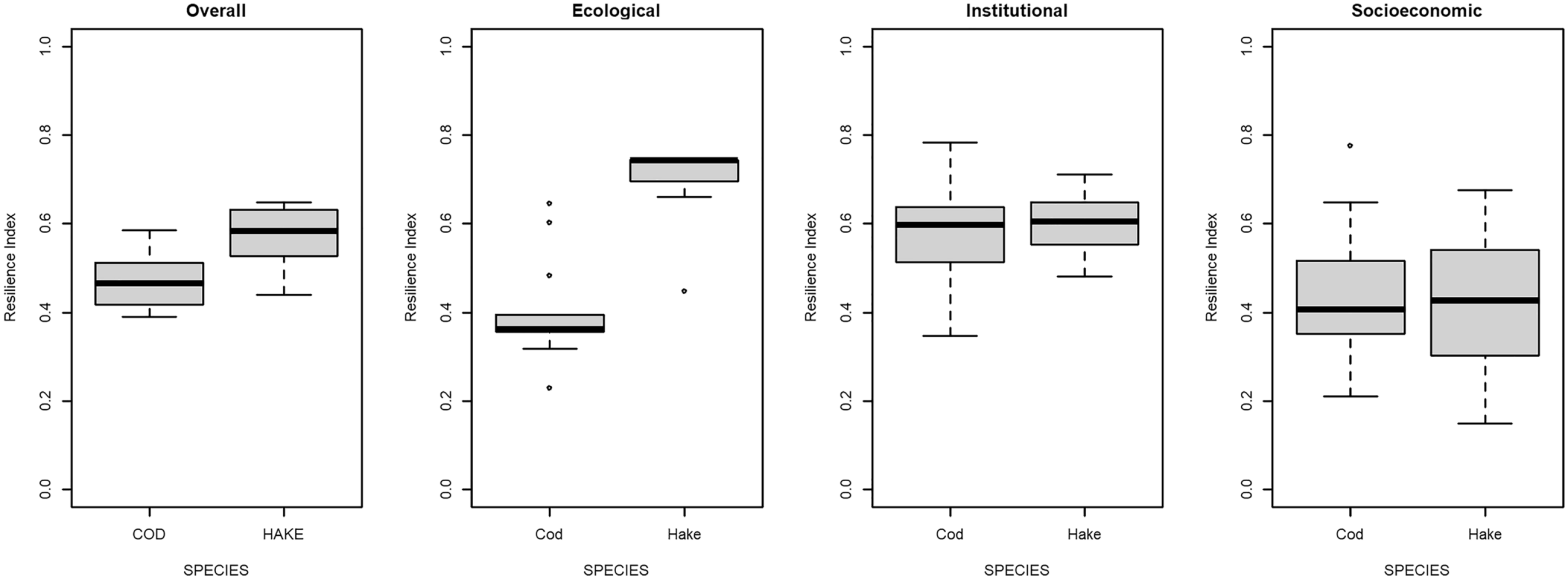
Boxplot for the overall, ecological, institutional, and socioeconomic resilience indexes per species (Cod and Hake).

**Figure 2:**
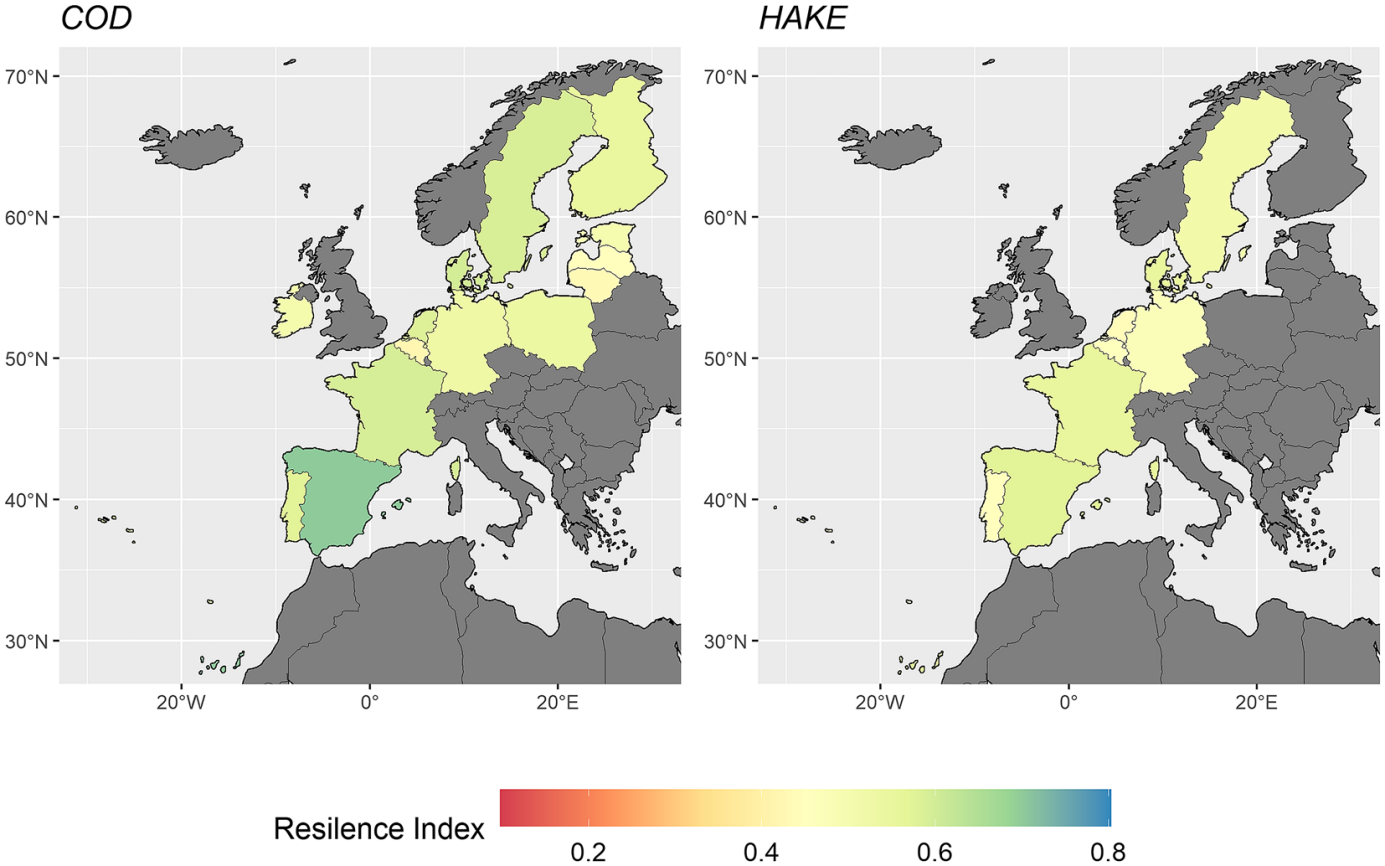
Resilience index results by species (Cod and Hake) and country. The maps were created using the free package “rnaturalearthdata” in R 4.0.3^68^.

Figure 1 shows the overall resilience index and its decomposition per dimension for cod and hake. Hake has a higher ecological and institutional resilience index than cod, while we do not observe significant differences between species in the socioeconomic dimension. As a consequence, the overall resilience index of hake is higher. These differences between species can be linked to some key factors. In the socioeconomic dimension, adaptive management and fleet mobility play a major role, where the low factor scores for countries reduces the resilience index (Fig. SI.3). In the institutional dimension, countries compliance with regulations (strength) and the number of organizations for co-management also limit the resilience performance of countries, as they score low values for both species (See Fig. SI.3). In the ecological dimension, two factors compromise resilience, abundance, and the overexploitation status of the stocks that countries fish (Fig. SI.3). Resilience index scores per country are mapped in Fig. 2, where overall scores do not differ greatly between countries, and the main differences across species take place mainly for Spain, France, and Sweden.

**Figure 3:**
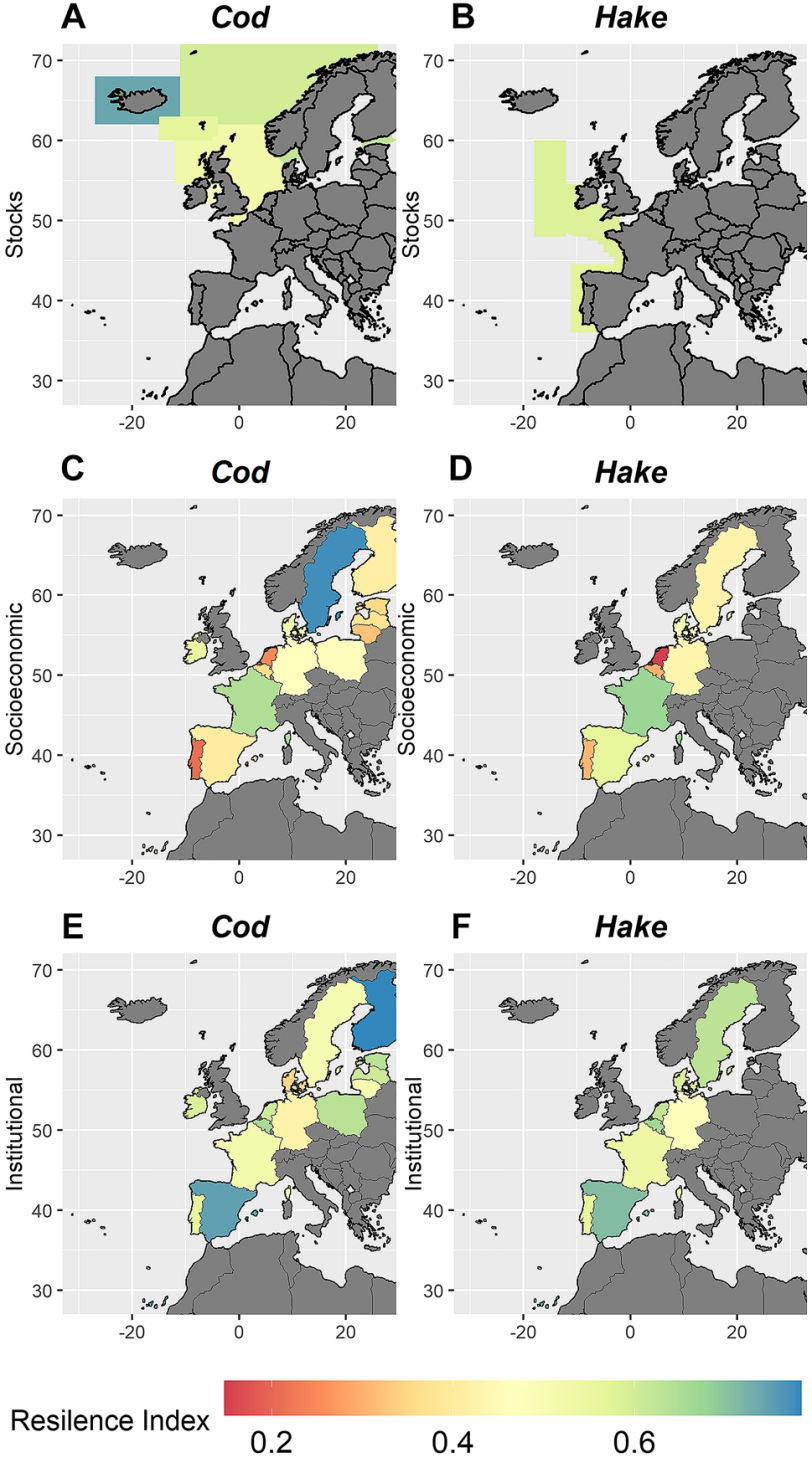
Results for the ecological, socioeconomic, and institutional resilience dimensions, per country and species. (**A** and **B**) represent ecologic, (**C** and **D**) socioeconomic, and (**E** and **F**) institutional dimensions. The maps created using the free package “rnaturalearthdata” in R 4.0.3^68^.

Figure 3 shows the resilience index per dimension. Ecological resilience for cod and hake is shown in the stock management areas of EU waters (Fig. 3 panels A, B). Only one stock of cod in Iceland has a high resilience value. For the socioeconomic resilience dimension, we find that countries’ resilience greatly differs, with Portugal and Belgium having the lower resilience index levels for both species. In both cases, the lower socioeconomic resilience values are due to a low fleet mobility and a low score of adaptive management, and due to their dependence on less resilient stocks (Fig. 3 panels C, D). In terms of institutional resilience, values are similar across species, with Spain and Finland having the greater resilience in the cod fishery (Fig. 3E) and Spain also having the highest resilience for the hake fishery (Fig. 3F). While Spain has high values on the co-management and property rights factors for institutional resilience, it holds the lowest value in institutional strength due to low compliance of fisheries regulations (strength factor) (Fig. SI.3). In the case of Finland, the high values in the institutional factors quotas and strength contrast to the lowest value on the co-management factor (Fig. SI.3).

**Figure 4:**
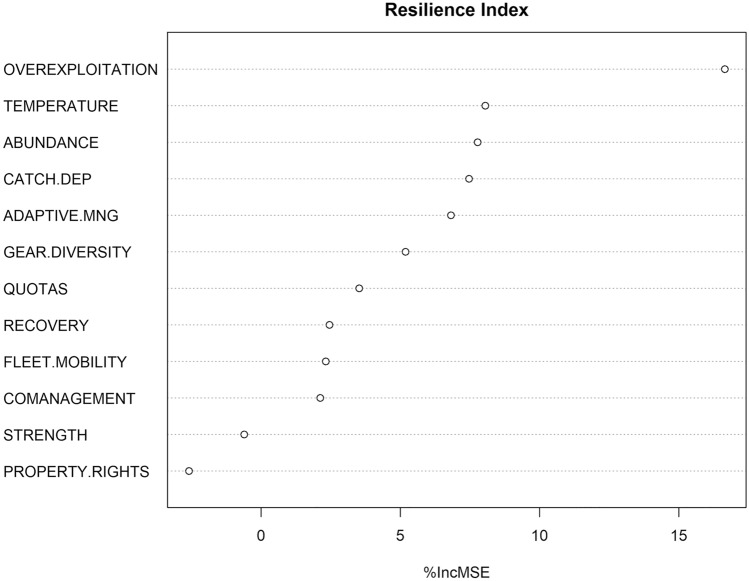
Random Forest results of the factors contributing to the resilience index.

In order to further explore which resilience factors are driving the results for the studied species, a random forest analysis of the resilience factors was carried out. Figure 4 shows that overexploitation is the single factor contributing the most to the final resilience index, with > 15% mean decrease accuracy. We interpret this finding as the importance of the status of the assessed stocks on the overall final resilience of countries. Other factors also influence resilience, but their contribution is lower. The temperature ranges of species (temperature factor), followed by their abundance, also affect resilience. The socioeconomic and institutional factors come next with catch dependency on species by countries and gear diversity having the higher contributions. Figure SI.8 shows the partial dependence and Pseudo R square of the random forest model.

Following Melnychuk et al.^31^, we compare our results with additional climate change and economic indices at the country level (Fig. SI.7), obtaining an expected positive relationship with the readiness to adapt index, and an inverse relationship to the vulnerability index (Fig. SI.7). Finally, the latitudinal effect on the resilience index was also explored, as stocks that shift poleward may influence how resilience is distributed across the latitude gradient. Hence, a GAM model applied to the resilience index including the latitude of stocks and countries, found only a small effect of latitude in the institutional resilience of countries, with greater resilience in lower latitudes for cod, and in lower and higher latitudes for hake. We conclude that further analyses are needed to better understand these latitudinal effects, as well as the role of other non-ecological factors such as institutions.

### Adaptation pathways for resilient fisheries in the EU

The present study proposes an indicator-based approach to develop a resilience index for marine commercial species under the impacts of climate change in the context of distributional shifts of stocks. We take into account ecological, socioeconomic and institutional factors to explore the resilience of European countries in terms of their hake and cod fisheries. We provide an operationalization of resilience that can further inform fisheries policy, where managing for resilience takes into consideration new factors, specially from the socioeconomic dimension, such as fishing mobility, or from the institutional one, such as property rights. This approach also considers the socioeconomic dimension with focus on dependency over overexploited stocks as well as on the gear and fleet diversity.

The social-ecological resilience estimates presented can have several possible implications for management which we summarize here within the context of climate resilient development pathways^52^. Following Werners et al.^53^, we contextualize three adaptation pathways for EU fisheries. First is the multi-stakeholder-oriented adaptation pathway^53^. In this realm, our results suggest that fisheries management could benefit from an improved institutional dimension in most EU countries, which is incorporated in our approach as the number of producer organizations that can contribute to co-management. Besides a strong organized fisheries system, this pathway would require a strong participation of stakeholders in decision making in the form of co-management, which is known to increase resilience and adaptive capacities under climate change^46,54,55^. Participation and organization in the fishery promote collaborative learning, adaptive planning, and adaptive capacity^53,56^. For EU fisheries there is a long way to go in order to improve participation and co-management, in a system that lacks transparency on decision making and is governed mostly top-down^57,58^.

The second adaptation pathway for EU fisheries is performance-threshold oriented^53^. The existing common fisheries policy (CFP) target for 2020 of managing all stocks at MSY has not been yet met^54^. Our study shows that this target is key for resilience as we find that overexploitation is the most important factor in constraining the fisheries resilience of countries. We propose this as a short-term priority for EU fisheries in order to address climate change impacts, and an urgent goal as management decisions still often surpass scientific advice^59^. In addition, we have also seen that adaptive management is key and constrains resilience in many countries. While we have focused on research and management investments as resilience factors, for this pathway near-term targets could include climate adaptive management^33^, which involves monitoring of climate change impacts and decision making that incorporates climate forecasts^54,60^. There is little evidence of climate adaptive management being implemented across countries and fisheries management bodies^61^, and EU fisheries policy could concentrate on this goal to meet short and longer-term adaptation needs.

Finally, the third adaptation policy pathway concentrates on transformation-oriented approaches^34,53^. Transformation involves structural changes in the fisheries system in order to manage impacts, when adaptation responses are not enough^24^. This pathway focuses on the longer term, accounting for the complexity of the system^53^, and involves potential changes to fishers’ livelihoods (i.e., diversification), as well as institutional and organizational transformations (i.e., food security and equity goals^62^). In our results, we have seen the differences in countries’ property rights systems with the indicator quota swapping. In the EU, quotas can be swapped at the country level in a process that involves fishers’ organizations^47^. This system often creates impacts in small scale fisheries and regional conflicts within and between countries^63^, and the degree to which it confers resilience in the fishery system is under debate. Another factor that we highlight here is fleet mobility. As stocks are expected to shift northwards in response to climate change, the impact on southern countries is likely to be stronger than in northern countries^54^. In this context, the mobility of European fisheries could be seen as a pivotal factor to consider for longer term adaptation. This socio-political variable reflects the history of the fleets and institutional international arrangements. As species shift northwards and the areas of distribution cross institutional borders^64,65^, new geopolitical conflicts can arise between countries, which possibly requires a transformational change in the management system.

Vulnerability and risk assessments^17,19^ are experiencing increasing interest from the scientific community as knowledge-transfer tools to decision makers. As for the resilience approach here proposed, these assessments transform raw ecological, biological, social, or economic data into indicators and composite indices that can be easily transferred to non-academics. Both vulnerability and risk assessments (the most recent approach), consider different dimensions of the system under study, (namely exposure, sensitivity, and adaptive capacity), to allocate the different indicators, similar as we have proposed here considering the institutional, socioeconomic, and ecological dimensions. Nevertheless, it has to be noted that the meanings of these dimensions, as well as the relationship between them, are not fixed and varied in recent years. The resilience approach proposed here meets the family of vulnerability assessments in the sense that it is created to assist decision and policy makers from an academic perspective and shares the fundamentals of its methodology. We believe, however, that the resilience assessment can be considered in parallel to vulnerability/risk estimates as it provides new insights on the systems of study, providing complementary information to the sensitivity and adaptive capacity dimensions that greatly informs adaptation policy pathways. Defining the limits of resilience as we propose could benefit new literature in this regard, avoiding the overlap of terms (e.g., susceptibility, adaptive capacity, robustness, etc.) and limiting the many different dimensions of vulnerability proposed all over the existing literature.

We conclude that taking a social-ecological resilience approach in fisheries can be an effective climate adaptation pathway and we have identified three complementary pathways that can help the system of EU fisheries meet this goal. While at the shorter-term, ending overfishing and improving the organization and participation of the fisheries stakeholders in decision making is key, longer-term adaptation requires climate adaptive management and a transformation of the access and allocation system. Our resilience index approach has helped us diagnose the status of the fisheries however, further factors that we have not been able to consider here can play an important role and should be considered in further analyses. These are livelihood diversification, participation in decision making and climate adaptive management. Further research could explore the index for the range of fisheries managed by a regional body in order to allow for species-level comparison of resilience performance of the fisheries, or overall country fisheries resilience by including all relevant stocks.

## Methods

The present research develops a methodology to measure resilience to climate change in EU fisheries. We focus on two marine commercial species and do the analysis at the stock level for the ecological dimension, and at the fishing country level for the socioeconomic and institutional dimensions. Methods are replicable and transferable to other stock managed species in the EU directly, and to other fisheries elsewhere by adapting the institutional factors to the regulatory frameworks at place.

To identify specific conditions that enhance resilience, we review the existing literature collecting variables that have been related to an increase in ecological, socioeconomic, or institutional resilience. We assess each variable and classify it according to our reference framework of potential resilience factors^29^. As a result, we derive our analysis from the set of resilience factors and establish three different dimensions, named ecological, socioeconomic, and institutional (Table 1).

For each of the factors identified in the literature, we select indicators that are fed by raw data (Table 2). Data collection is described in Table 4.1 of the SI, and includes species characteristics (i.e., thermal range, distribution area), stock dynamics (i.e., biomass, fishing mortality, recruitment), and fishery characteristics (i.e., economic dependence, regulations). Most of the ecological indicators come from the species stocks’ assessments, where we estimated linear trends for the historic (1950–2010) and more recent (1980–2010) spawning stock biomass (SSB), fishing mortality (M) and recruitment (R) to capture the dynamics of the stocks over time, corrected by the stock specific average biomass, mortality and recruitment for comparability across stocks and species (see Table 2 for a correspondence between indicators and factors). We also collect information on the stock status and recovery potential that is incorporated in the factors overexploitation and recovery time. Species specific information are entered in the factors distribution area and temperature range of the species in the form of distributional ranges (km^2^, Table 2) and thermal tolerance limits (°C, Table 2). Specific information on the fishing dynamics of countries is collected by means of past catches in the spatial management areas of the ICES, which the EU uses for catch allocation policy. Hence, we compare past fishing areas with current fishing areas in the factor fleet mobility, as well as the number of areas a fishing country has access to. Dependency of fishing countries is calculated as the proportion of catch from the total catch of each country in catch dependency, using fishing statistics by country. Regulation and fishery characteristics are obtained from annual policy of total allowable catch (quotas) and published information on quota exchange (property rights). The factor property rights is positive with resilience (Table 2, column Direction), as we consider that countries earning money from selling their quotas are able to diversify their catch and fishing activities when the fishery is not profitable or when the target species move to different areas. The co-management factor is measured as the number of fisher organizations per country assuming that the higher the number of associations, the higher the diversity of opinions represented and the transmission of fisher demands and local knowledge to managers (Table 2). The full list of indicators with the methodology used for collecting the data from existing databases and reports, is available in the SI.

To combine the set of indicators we rely on previous studies employing indexes for fisheries management^31^, vulnerability assessments^17^ and fisheries social-ecological systems^32^. Following Leslie et al.^32^ and Burgass et al.^66^, we normalize all the factors per dimension to a range of 0–1 using normalization values specified in the SI^1,26,67^. We do this regardless of whether the primary data used to develop the indicator were qualitative or quantitative, using Eq. 1 in the SI (section SI 2.1), reversed for factors negatively affecting resilience (i.e., Overexploitation). Following Cinner et al.^17^ and Melynchuk et al.^31^ we check our indicators correlation in order to avoid problems in the aggregation of the resilience factors. We estimate the correlation matrices for the ecological, socioeconomic, and institutional indicators (Fig. SI.2—correlation matrices). Indicators that are correlated with a coefficient over 0.75 are dropped from the subsequent analysis to avoid double counting and inflation of the factors and dimensions in the final index^17^. The aggregation process is a stepwise approach where indicators are averaged to obtain factors, which are averaged into dimensions, and finally, averaged into the composite resilience index. No weights are used in any of the steps, but the approach could be adapted to expert-based weighting. See SI for further information.

Generalized additive models (GAM) with quasibinomial family and logit link were applied to analyze whether the resilience scores depend on latitude. GAM fitting was conducted with the ‘mgcv’ package of R^68,69^. We also conducted a Random Forest model for exploring which factors have more influence on the resilience index value. We use the “randomForest” package (version 4.6-14^17^) in R (see Sect. 8 in the SI).

## Supplementary Information

Supplementary Information 1.
