# Supplementary material for: Assessing countries’ social-ecological resilience to shifting marine commercial species
Source: Sci Rep. 2021 Nov 25;11:22926. doi: 10.1038/s41598-021-02328-6 (PMC8617226; doi:10.1038/s41598-021-02328-6)
Supplement: Supplementary file 1 — Supplementary Information 1. [file 41598_2021_2328_MOESM1_ESM.pdf]

Supplementary Information

Assessing countries' social-ecological resilience to shifting marine commercial species

Elena Ojea*,[1], Elena Fontán[1], Isabel Fuentes-Santos[2] and Juan Bueno-Pardo[1]

[1] Future Oceans Lab, CIM-Universidade de Vigo (Spain)
[2] Instituto Español de Oceanograía-Vigo (Vigo, Spain)

Contents

Supplementary Information 1
1 Approach 3
1.1 Potential resilience factors 3
1.2. Resilience factors measurement 5
1.3 Data analysis 6
2. Standardization of indicators 7
2.1 Normalization 7
2.2. Correlation analysis 7
2.3. Index aggregation 8
3. Calculation of index values 10
3.1 Ecological indicators and factors 10
Area (E1) 10
Abundance (E2) 11
Temperature (E3) 13
Overexploitation (E4) 14
Recovery (E5) 17
Ecological factors summary 18
4. Socioeconomic indicators and factors 21
Gear diversity (S1) 21
Fleet mobility (S2) 21
Catch dependency (S3) 23
Adaptive management (S4) 26
Social factors summary 27
5. Institutional Indicators and Factors 28
Co-management (I1) 28
Property rights (I2) 29
Quotas (I3) 30
Strength (I4) 31
Institutional factors summary 32
6. Resilience factors summary 33
7. Index performance 35
8. Additional analyses 37
9. Bibliography 40

1 Approach

Figure SI.1 shows the steps of the methodology that are followed to build the Fisheries Resilience Index. We review the literature to identify potential resilience factors and we classify these into the three dimensions of the index: ecological, socioeconomic, and institutional. Then, we explore the data available for measuring these factors per stock, per species and per country. We identify several indicators to quantitatively measure the potential resilience factors. The work with the indicators starts with data analysis to measure the indicators, by means of plots and linear models. Then we perform a correlation analysis to exclude duplicated information from our indicators (section 1.2), we normalize the indicators, and we aggregate the indicators into factors. Finally, factors are aggregated into dimensions and are averaged in the final Fisheries Resilience Index. What follows is a more detailed explanation on each step of the methodology. A detailed description of the data used and results from the construction of the indicators is available in the section 3: "Calculation of index values".

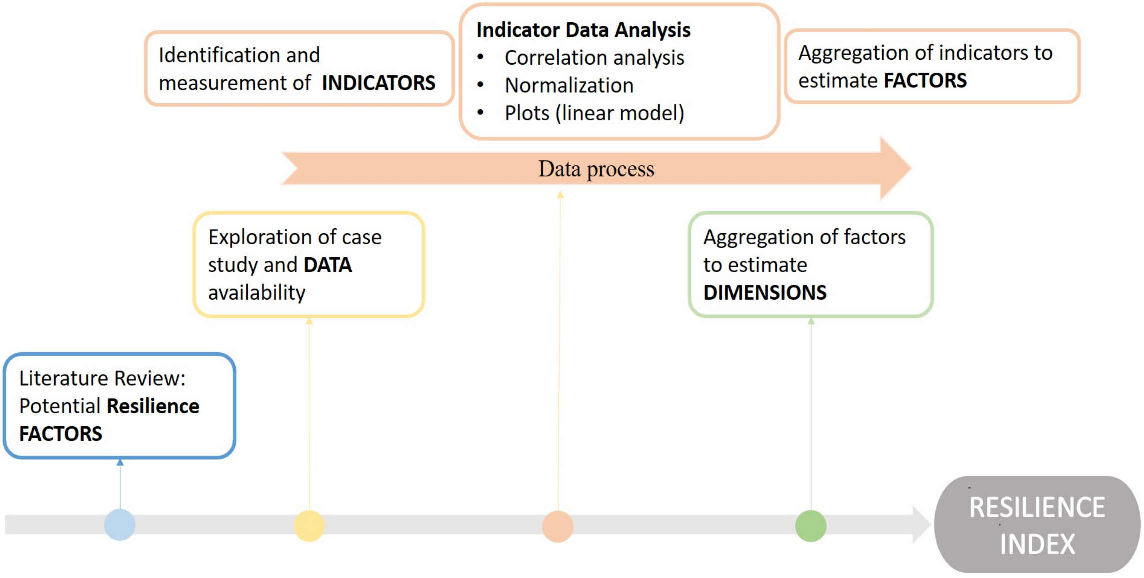

Figure SI.1. Steps in the methodological approach and data process for the construction of the fisheries resilience index.

1.1 Potential resilience factors

A literature review was conducted to identify specific settings and conditions on a fishery that enhances resilience. We refer to these settings and conditions as "resilience factors". Previous work has identified a set of resilience factors for fisheries and discuss the role of fisheries regulatory regimes in promoting or not these factors [1]. This effort has been expanded by looking at a broader set of resilience factors from the literature [2, 3, 4, 5], and classifying these factors into three resilience dimensions: ecological, socioeconomic, and institutional. Table SI.1 summarizes the collected evidence together with the description of each indicator (see Table 2 of the main text for a correspondence between the factors and indicators used), the sources in the literature for the statement, and an explanation of why the indicator may increase resilience.

Table SI.1. Methods, measurement, units, and sources for the indicators selected.

	INDICATORS	METHODS	MEASURE	UNITS	SOURCE
ECOLOGICAL	*Area2006*<br>*Area2100*	Relative change of the species potential distribution area in 2100 relative to 2006 (Area2100-Area2006)/Area2006	Global projections for 2006 and 2100 based on climate envelop models and scenario RCP8.5	proportion	García-Molinos et al., 2016
	SSB (Spawning Stock Biomass) historic	Coefficient of the linear model for the historic trend of SSB (1950-2018, varies per stock assessment)	Slope for the linear regression of SSB divided by average SSB of stock	dimensionless	RAM Legacy Stock Assessment Database. (2020). RAM Legacy Stock Assessment Database v4.491 (Version v4.491) [Data set]. Zenodo. http://doi.org/10.5281/zenodo.3676088
	SSB (Spawning Stock Biomass) 1980-present	Coefficient of the linear model for the SSB in the last 38 years (1980-2018, varies per stock assessment)	Slope for the linear regression of SSB (1980-2018) , divided by average SSB of stock	dimensionless	
	Ftrend (mortality per fishing) *	Coefficient of the linear model of F for the historic trend (1950-2018, varies per stock assessment)	Slope for the linear regression of catches (1950-2018), , divided by average F of stock	dimensionless	
	Rtrend (recruitment)	Coefficient of the linear model of R for the historic trend (1950-2018, varies per stock assessment)	Slope for the linear regression of catches (1950-2018), , divided by average R of stock	dimensionless	
	Trange	Preferred temperature range for the species at the 25 and 75 percentiles	Tª (q75)- Tª (q25)	ºC	Cheung et al., 2013
	T50	Medium preferred temperature of occurrence for species (50 percentile)	Tª (q50)	ºC	
	OverMSY*	Relation of B/Bmsy and F/Fmsy from RAM Legacy, last 10 years of stock data	(B/Bmsy) /(F/Fmsy)	proportion	RAM Legacy Stock Assessment Database. (2020). RAM Legacy Stock Assessment Database v4.491 (Version v4.491) [Data set]. Zenodo. http://doi.org/10.5281/zenodo.3676088
	Status	Most recent position in Kobe plot using SSB/SSBlimit and F/Flimit from RAM legacy data on stock	0: upper left, 1: upper right, 2: down left, 3:down right (sustainable)	dimensionless	
	Recovery*	The number of years the stock needs to go from SSBlimit to 1.5*SSBlimit	Year (stock > 1.5*SSBlimit) - Year(stock < SSBlimit)	years	Neubawer et al. 2013
	**INDICATOR**	**METHODS**	**MEASURE**	**UNITS**	**SOURCE**
SOCIOECONOMIC	SPgear	Number of different gears used to fish the species per country (1950-2018)	Number of gears used per species and by country	number	Sea around Us<br>Pauly D., Zeller D., Palomares M.L.D. (Editors), 2020. Sea Around Us Concepts, Design and Data (seaaroundus.org).
	ICESAreas5	Number of ICES areas where a country fishes a stock across a 5 year period	Average number of ICES areas per country, from 2006 to 2010	number	Historical Nominal Catches 1950-2010 in the Major Fishing Area 27
	ICESAreasEU	The change in the number of areas a country has quota over a stock before and after the EU fisheries policy	Average ICES areas before 1993 - Average ICES areas after 1993 per country	number	Historical Nominal Catches 1950-2010 in the Major Fishing Area 27
	Stock dependency per species*	Proportion of the annual catches of a species coming from a specific stock relative to all species catch in the country. Averaged across 5 years	Catch stock/Catch species per country in the period 2006-2010	proportion	Historical Nominal Catches 1950-2010 in the Major Fishing Area 27<br>RAM legacy (Ricard et al., 2012)
	Stock dependency total*	Proportion of the annual catches of a species coming from a specific stock relative to total country catches. Averaged across 5 years	Catch stock/Catch totals per country in the period 2006-2010	proportion	
	Catch trends	Historic trend of catches per specie and country. The linear model slope is normalized by the average of the total catches of the period of each specie.	Slope for the linear regression of catches (1950-2010)	million tonnes/yr	Historical Nominal Catches 1950-2010 in the Major Fishing Area 27
	Research	Data from *SeaAroundUs* webpage. They include investment in research and management ("good subsides")	Subsides per country	million euro	Sumaila UR, Khan A, Dyck A, Watson R, Munro R, Tydemers P and Pauly D (2010) A bottom-up re-estimation of global fisheries
	Management				

					subsidies. Journal of Bioeconomics 12: 201-225.
	**INDICATOR**	**METHODS**	**MEASURE**	**UNITS**	**SOURCE**
INSTITUTIONAL	N.organizations	Number of Producer Organizations per country in 2017	Producer organizations per country 2017	Number of organizations	Producer organizations in fisheries as listed by the EU CFP (List of recognised producer organisations and associations of producer organisations)
	Swaps	Total value of quota swaps per country 2000-2006	Money earned by country from quota exchanges from 2000 to 2006, as obtained from Andersen et al., 2009 Table 8. Net quota exchanges at member state level measured in value. Total 2000-2006.	million euro	Andersen et al., 2009 (Andersen, J. L., Nielsen, M., & Lindebo, E. (2009). Economic gains of liberalising access to fishing quotas within the European Union. *Marine Policy*, *33*(3), 497-503.)
	AboveTAC*	Catches of a stock over the established Total Allowable catches (2015)	(TAC – ICES advice)	tones	Carpenter et. al. (2016) SSMM (Carpenter, G., Kleinjans, R., Villasante, S., & O'Leary, B. C. (2016). Landing the blame: The influence of EU Member States on quota setting. *Marine Policy*, *64*, 9-15.)
	Compliance*	IUU Fishing Index (2018)	Level of compliance with fishing regulations and effort against Illegal Unreported unregulated Fishing.	dimensionless	Macfadyen, G., Hosch, G., Kaysser, N. and Tagziria, L., 2019. The IUU Fishing Index, 2019. Poseidon Aquatic Resource Management Limited and the Global Initiative Against Transnational Organized Crime.

*Negative relation to resilience

1.2. Resilience factors measurement

In order to operationalize the framework, we consider hake and cod stocks in European Atlantic waters under the Total Allowable Catches (TAC) system. We only include European Union countries with at least one year catch records of at least one of the stocks in the period of 1950 – 2015 (Historical Nominal Catches 1950-2010 https://www.ices.dk/data/dataset-collections/Pages/Fish-catch-and-stock-assessment.aspx). These countries are Belgium, Denmark, Germany, Estonia, Ireland, Spain, France, Latvia, Lithuania, Netherlands, Poland, Portugal, Finland, and Sweden. We exclude those countries called "third part", that are outside of the EU, named Norway, Iceland, or UK. The stocks of the two species are identified and information was obtained from the RAM legacy database [6]. Table SI.2 shows the list of stocks used for the analysis. For each factor, the selection of indicators and how to measure them is based on previous studies employing indices for fisheries management [7], vulnerability assessments [8] and fisheries socio-ecological systems [9]. We also rely on previous resilience approaches that looked at one specific component such as recovery time [4] or economic resilience [3]. Once the list of potential indicators and the methodology is defined, we collect the data from existing databases and reports, mainly at the European level. Ecological data was mainly obtained from the RAM Legacy database [6], the ICES Historical Nominal Catches (http://www.ices.dk/marine-data/dataset-collections/Pages/Fish-catch-and-stock-assessment.aspx) was downloaded for the last time on November 2020, and other databases and references were available in the Eurostat portal [12] and in Fishbase [13]. Social and institutional data was obtained from Sea Around Us [14], Eurostat (https://ec.europa.eu/eurostat/data/database), the United Nations Development Program (UNDP, https://www.undp.org/) and the Food and Agriculture Organization of the United Nations (FAO, http://www.fao.org/home/en/), among others (see Table SI.1 for data sources). These indicators could be further developed upon data availability in the specific stock, species, or country in future applications.

Table SI.2. List of Stocks used to operationalize resilience (RAM Legacy database)

Assessment ID	Assessor ID	Stock ID	Stock area	Recorder
AFWG-CODNEAR-1943-2018-ICESIMP2018	AFWG	CODNEAR	Atlantic cod North-East Arctic	ICESIMP2018
AFWG-CODNEARNCW-1984-2017-ICESIMP2018	AFWG	CODNEARNCW	Atlantic cod North-East Arctic (Norwegian coastal waters)	ICESIMP2018
NWWG-CODFAPL-1958-2018-ICESIMP2018	NWWG	CODFAPL	Atlantic cod Faroe Plateau	ICESIMP2018
NWWG-CODICE-1952-2018-ICE-SIMP2018	NWWG	CODICE	Atlantic cod Iceland Grounds	ICESIMP2018
WGBFAS-CODBA2532-1965-2017-ICESIMP2018	WGBFAS	CODBA2532	Atlantic cod Eastern Baltic	ICESIMP2018
WGBFAS-CODKAT-1996-2018-ICESIMP2018	WGBFAS	CODKAT	Atlantic cod Kattegat	ICESIMP2018
WGCSE-CODIS-1968-2017-ICE-SIMP2018	WGCSE	CODIS	Atlantic cod Irish Sea	ICESIMP2018
WGCSE-CODVIa-1980-2017-ICE-SIMP2018	WGCSE	CODVIa	Atlantic cod West of Scotland	ICESIMP2018
WGNSSK-CODIIIaW-IV-VIId-1962-2018-ICESIMP2018	WGNSSK	CODIIIaW-IV-VIId	Atlantic cod ICES 3a(west)-4-7d	ICESIMP2018
WGBIE-HAKENRTN-1978-2018-ICESIMP2018	WGBIE	HAKENRTN	Hake ICES 3a-4-6-7-8abd	ICESIMP2018
WGBIE-HAKESOTH-1982-2018-ICESIMP2018	WGBIE	HAKESOTH	Hake ICES 8c-9a	ICESIMP2018

1.3 Data analysis

The R Statistical software [21] was used to analyze data and produce the figures of the manuscript, including the maps and graphics. We also perform random forest [12] over the final index values to explore what factors have greater impact on the index values and generalized additive models (GAM) [22] to explore latitudinal effects on the resilience index. The code used for the analysis is available in a Github repository: https://github.com/future-oceanslab/Fisheries-Resilience-Index.

2. Standardization of indicators

2.1 Normalization

All the indicators in this document (x) were standardized (x') between 0 and 1 following the general formula:

$$x' = \frac{x - min(x)}{max(x) - min(x)} \quad \text{Eq. 1}$$

where *min(x)* represents the minimum theoretical value of x, and *max(x)* its maximum theoretical value. When an indicator is referred to be standardized within the sample, it means that the minimum and maximum values min(x) and max(x) are taken from the observed values.

2.2. Correlation analysis

Following [7] and [8] we check our indicators correlation in order to avoid problems in adding up the resilience factors. We estimate the correlation matrices for the ecological, socioeconomic and institutional indicators in Figure SI.2. Indicators that are correlated with a coefficient over 0.76 are dropped from the subsequent analysis as to double counting and inflation of the factors and dimensions in the final index [8]. From the correlation results, only ecological indicators have correlation scores over our threshold, therefore institutional and social factors are computed using all indicators. In the case of ecological factors, we drop the Area indicator as it is highly correlated with Temperature. We also drop SSBhistoric and Rtrend as they correlate with SSBrecent and Ftrend.

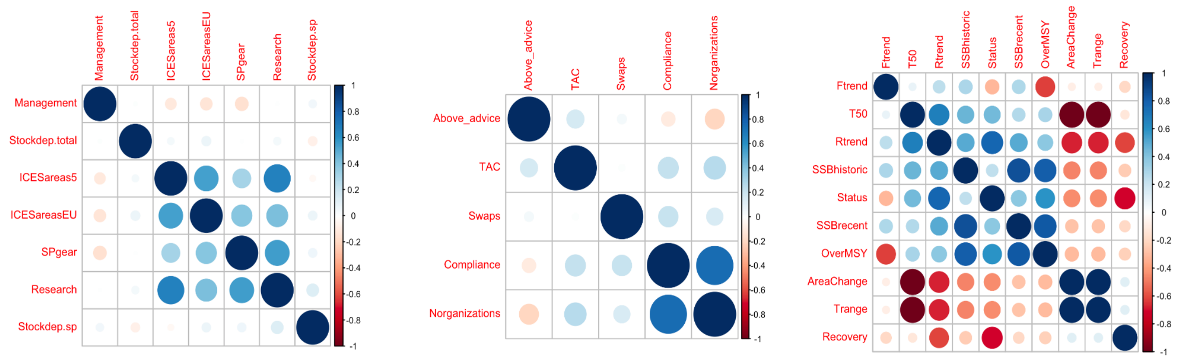

Figure SI.2. Correlation matrices among indicators for the ecological (left), socioeconomic (centre) and institutional (right) dimensions of the resilience index.

2.3. Index aggregation

After performing the correlation analysis and dropping off highly correlated indicators, we combine the remaining set of indicators into factors and dimensions. For each level of aggregation, we use simple averages to obtain the values of the factors and dimensions at the country and species level for ecological, socioeconomic, and institutional resilience. All indicators, factors and dimensions are weightless. Results for the country and species factors are shown in Figure SI.3. Factors are also averaged across dimensions, as shown in Figures SI.4 and mapped in Figure SI.5.

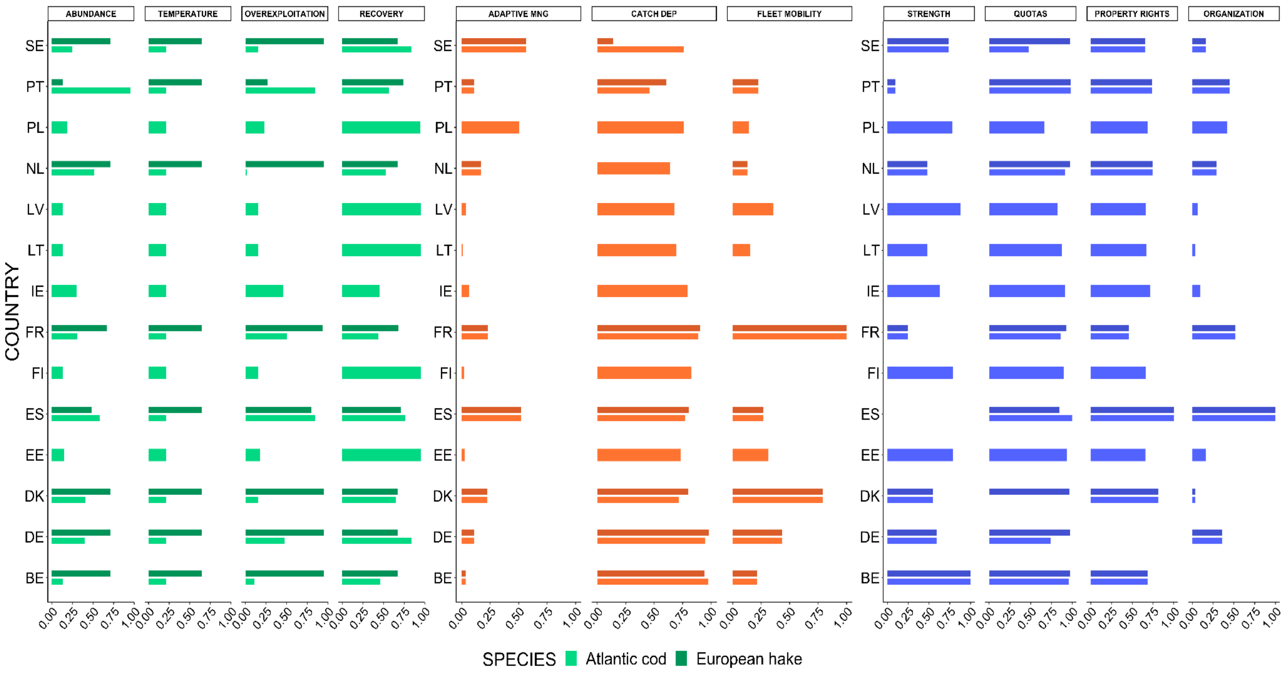

Figure SI.3. Factor values for the two species and per country.

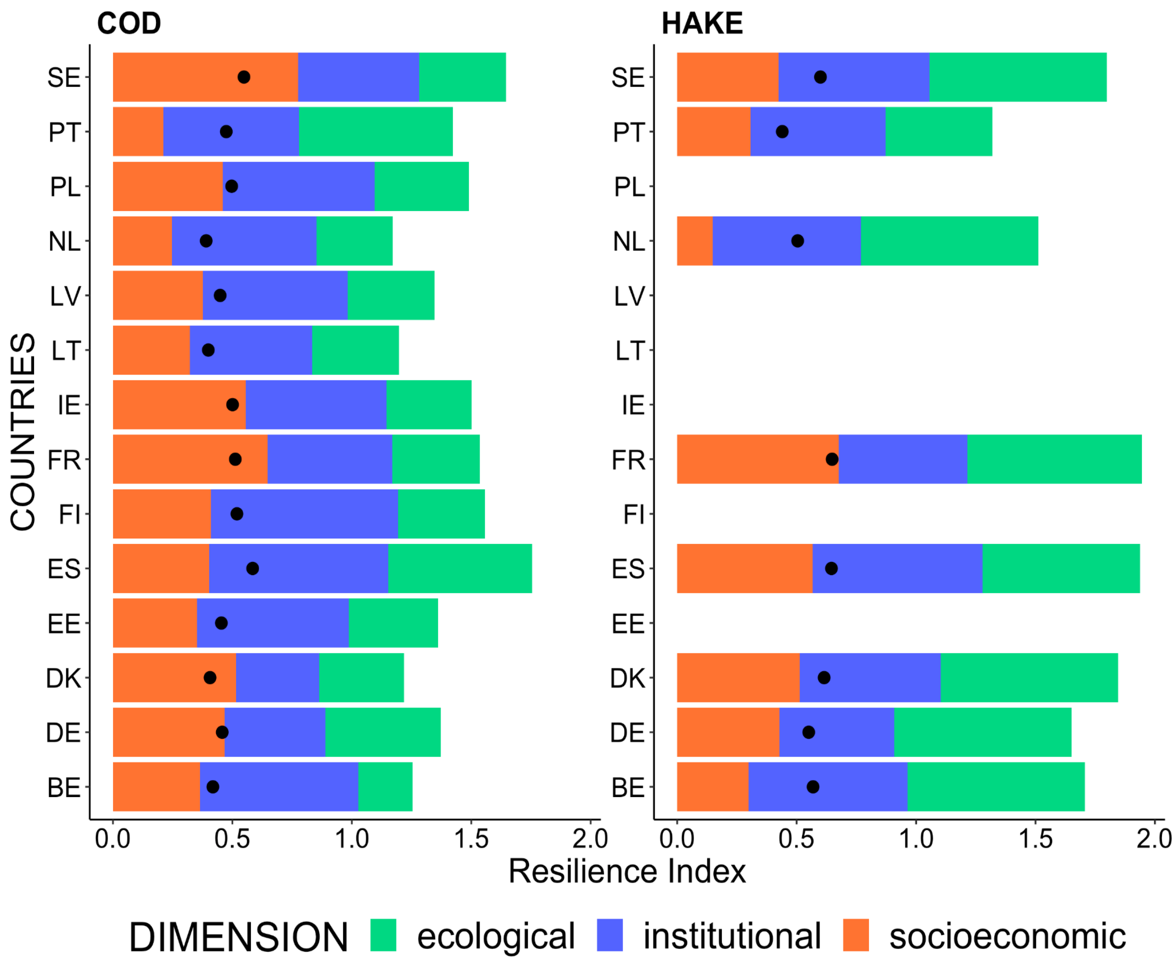

Figure SI.4. Dimension values for the two species and per country.

3. Calculation of index values

3.1 Ecological indicators and factors

Area (E1)

This factor captures the expected change in the species' distribution area. Using climate envelope models [13] the projected distribution area of the species was obtained for 2006 and 2100, under climate change scenario RCP 8.5. The factor was then calculated using the formula*:

$$AREA = \frac{AREA_{100} - AREA_{06}}{AREA_{06}} \qquad \text{Eq. 2}$$

The factor was standardized between 0 and 1 assuming a minimum value of -1 (100% area decrease) and +1 (100% area increase) following Eq. 1 (Table SI3).

Table SI3. Area indicators and factor
.

Species	Area 2006	Area 2100	AREA*	Standardized AREA [0 - 1]	AREA*
Hake	5918472	6210615	0.05	0.52	0.05
Cod	7014930	7868484	0.12	0.56	0.12

*This factor is dropped from the analysis due to high correlation with Temperature.

Abundance (E2)

The abundance of the stocks was analyzed considering three variables: standing stock biomass (SSB), recruitment (R) and fishing mortality (F). A linear model was constructed where the coefficient for the slope (β) of each variable against time was used as a measure of the annual change of each indicator (Table SI.4). In the case of SSB, two different time slices were considered: "historic" between 1950 and 2010, and "recent" between 1980 and 2010. To make all stocks across species comparable, these changes were divided by the average of the stock between 1980 and 2010, according to Eq. 3. Raw data were obtained from the RAM legacy stock assessment database (2020) (http://doi.org/10.5281/zenodo.3676088).

$$Indicator = \frac{\beta}{stock\ average\ 1980-2010} \qquad \text{Eq. 3}$$

Table SI.4. Value of the coefficients ($\boldsymbol{\beta}$) from the linear models of SSB historic (1950-2010), SSB recent (1980-2010), R and F in RAM legacy.

Stock	β SSB historic	β SSB recent	β R	β F	SSB average	R average	F average
**CODNEAR**	15436	50925	-10130000	0.0001	813051.3	578611100	0.619
**CODNEARNCW**	-1887.4	-1887.4	-0.04	-0.0082	27552.38	1.00	0.323
**CODFAPL**	-889.4	-1547.7	-197174	0.0020	42874.36	15425530	0.579
**CODICE**	-3686	8496	-1151990	-0.0009	256794.9	153450000	0.569
**CODBA2532**	-6058	-15166	-7356000	-0.0040	225250	182531200	0.853
**CODKAT**	-0.06327	-0.06327	-0.05	-0.0402	0.9998182	1.00	1.000
**CODIS**	-333.9	-354.55	-1732438	0.0005	6641.132	27300770	0.906
**CODVIa**	-960.57	-960.57	-672345	0.0038	225250	11068650	0.906
**CODIIIaW**	-2838.9	-1085.6	-18380000	-0.0021	98135.9	461510500	0.831
**HAKENRTN**	5617	4892	42160	-0.0124	98966.67	358487179	0.737
**HAKESOTH**	-567.4	-567.4	733600	0.0064	17838.65	79467568	0.765

The normalization of the indicators between 0 and 1 was carried out for all stocks within the sample (Table SI5). Finally, the Abundance factor is computed as the mean of the four normalized indicators according to Eq. 4:

$$ABUNDANCE = \frac{SSB_{recent} + SSB_{historic} + F' + R'}{4} \qquad \text{Eq. 4}$$

Table SI5. Normalization of Abundance indicators and Abundance factor calculation*.

Species	Stock	SSB hist indicator*	SSB recent indicator	R indicator*	F indicator	SSB hist norm.*	SSB recent norm.	R norm*.	F norm.	ABUNDANCE
Cod	CODNEAR	0.019	0.063	-0.018	0.000	0.698	1.000	0.632	0.169	0.584
Cod	CODNEARNCW	-0.069	-0.069	-0.040	-0.025	0.000	0.000	0.323	0.695	0.347
Cod	CODFAPL	-0.021	-0.036	-0.013	0.003	0.381	0.247	0.697	0.101	0.174
Cod	CODICE	-0.014	0.033	-0.008	-0.002	0.432	0.775	0.770	0.205	0.490
Cod	CODBA2532	-0.027	-0.067	-0.040	-0.005	0.332	0.009	0.319	0.269	0.139
Cod	CODKAT	-0.063	-0.063	-0.050	-0.040	0.042	0.040	0.185	1.000	0.520

Cod	CODIS	-0.050	-0.053	-0.063	0.001	0.145	0.115	0.000	0.161	0.138
Cod	CODVIa	-0.004	-0.004	-0.061	0.004	0.513	0.490	0.037	0.086	0.288
Cod	CODIIIaW	-0.029	-0.011	-0.040	-0.003	0.316	0.438	0.325	0.224	0.331
Hake	HAKENRTN	0.057	0.049	0.000	-0.017	1.000	0.899	0.875	0.519	0.709
Hake	HAKESOTH	-0.032	-0.032	0.009	0.008	0.293	0.280	1.000	0.000	0.140

*Indicators dropped due to correlation, not used in factor average.

Temperature (E3)

The temperature factor was calculated for each species considering the 50th percentile of their average thermal preference using data from Cheung et al. (2013) [9] (Table SI6). The factor was then standardized using Eq. 1, between 0 and 1, considering the 2nd and 98th percentiles as minimum and maximum values respectively*.

Table SI6. Data and normalization of the average thermal preference of each species. Temperatures are shown in ºC.

Species	Temperature 50th percentile	Temperature 2nd percentile	Temperature 98th percentile	Temperature 50th norm.	TEMPERATURE*
EUROPEAN HAKE	18	0	28	0.643	0.643
ATLANTIC COD	6	0	28	0.214	0.214

Overexploitation (E4)

Factor overexploitation was composed of two indicators: status and OverMSY. For the Status indicator, standing stock biomass (SSB) and fishing mortality (F) data were used to analyze the historical status of the fishery. We use a Kobe-type plot displaying SSB/SSBlimit and F/Flimit. The indicator value results from this plot, where we look at the position of the species in the last year of data within the plot. In this respect, 0: upper left; 1: upper right; 2: down left; 3: down right. (Fig. SI.5 for cod; Fig. SI.6 for hake). Raw data were obtained from the RAM legacy stock assessment database (2020) (http://doi.org/10.5281/zenodo.3676088).

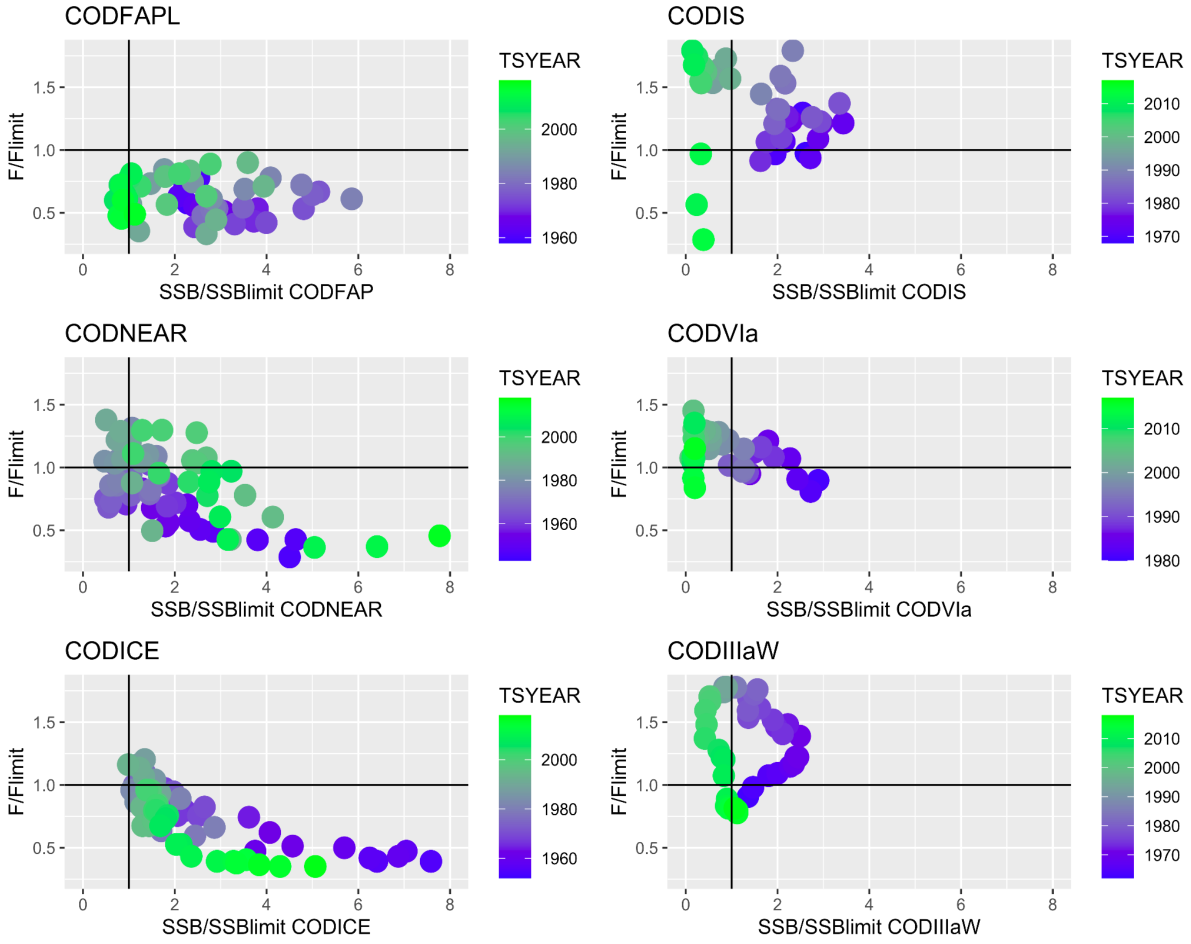

Figure SI.5. Kobe-type plot limited at SSB/SSBlimit and F/Flimit for cod stocks.

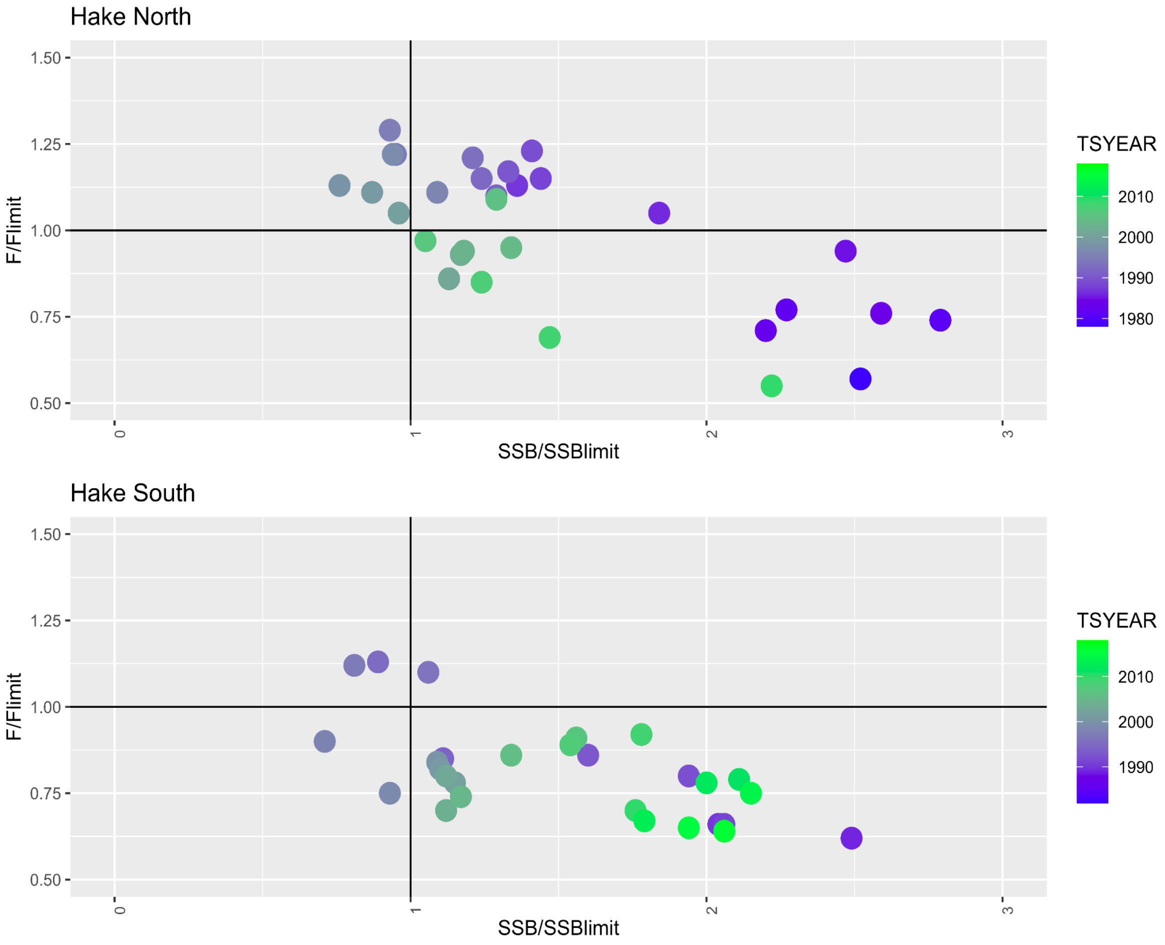

Figure SI.6. Kobe-type plot limited at SSB/SSBlimit and F/Flimit for hake stocks.

OverMSY is the difference between the observed catch (F) and the MSY (FMSY) estimate for the stock in 2012 as resulting from the model by Costello et al., 2016 (Eq. 5).

$$OverMSY = (F \,/\, \mathrm{F}MSY) \qquad \text{Eq. 5}$$

Note that both the Status and OverMSY indicators have opposite directions for resilience, while Status is positively related, OverMSY is negative. OverMSY is normalized using the inverse normalization formula, so it can be aggregated in the computation of the factor Overexploitation as follows*:

$$OVEREXPLOITATION = (Status + OverMSY) \,/\, 2 \qquad \text{Eq. 6}$$

Table SI7. Overexploitation indicators, normalization and factor

Stock	Status	Status norm.	OverMSY	OverMSY norm. (inverse)	OVEREX-PLOITATION
**CODNEAR**	3	1	5.1167	0	0.500
**CODNEARNCW**					
**CODFAPL**	2	0.75	0.2727	0.951	0.809
**CODICE**	3	1			1
**CODBA2532**			0.7489	0.857	0.857
**CODKAT**					
**CODIS**	2	0.5	1.3019	0.749	0.708
**CODVIa**	0	0	0.0219	1	0.500
**CODIIIaW**	2	0.5	0.4189	0.922	0.794
**HAKENRTN**	3	1	5.0269	0.018	0.509
**HAKESOTH**	3	1	0.4272	0.92	0.960

Recovery (E5)

Recovery time is observed in the time series data for each stock (ICES advice 2016) as the number of years between a stock going under the SSB limit established by ICES ($SSBlimit$) and going back to $1.5 * SSBlimit$. There are two stocks that never go back to $1.5 * SSBlimit$ during the time series, so for these two stocks we assume the largest recovery time observed in our sample (25 years). The recovery factor is based on one single indicator, Recovery, which is normalized considering the 2% and 98% of their sample as minimum and maximum respectively (Table SI.8).

Table SI.8. Recovery indicator, normalization, and factor.

STOCK	***recovery***	***recovery 2%***	***recovery 98%***	***RECOVERY***
CODNEAR	11.00	1.00	43.34	0.76
CODNEARNCW	18.00	1.00	43.34	0.60
CODFAPL	12.00	1.00	43.34	0.74
CODICE	4.00	1.00	43.34	0.93
CODBA2532	3.00	1.00	43.34	0.95
CODKAT	21.00	1.00	43.34	0.53
CODIS	24.00	1.00	43.34	0.46
CODVIa	25.00	1.00	43.34	0.43
CODIIIaW	25.00	1.00	43.34	0.43
HAKENRTN	15.00	1.00	43.34	0.67
HAKESOTH	12.00	1.00	43.34	0.74

Ecological factors summary

Table SI.9. Ecological factors per stock.

STOCK	*ABUNDANCE*	*TEMPERATURE*	*OVEREXPLOITATION*	*RECOVERY*
CODNEAR	0.584	0.545	0.500	0.764
CODNEARNCW	0.347	0.545	-	0.598
CODFAPL	0.174	0.545	0.809	0.740
CODICE	0.490	0.545	1.000	0.929
CODBA2532	0.139	0.545	0.857	0.953
CODKAT	0.520	0.545	-	0.528
CODIS	0.138	0.545	0.708	0.457
CODVIa	0.288	0.545	0.500	0.433
CODIIIaW	0.331	0.545	0.794	0.433
HAKENRTN	0.709	0.455	0.509	0.669
HAKESOTH	0.140	0.455	0.960	0.740

Ecological factors per stock are converted to factors per country based on whether the stock is being fished by the EU member country, based on the data from ICES Historical Nominal Catches 1950-2010 (https://gis.ices.dk/geonetwork/srv/api/records/c6ab8e49-324d-4940-9e9c-0281faa65310) (Table SI.8). The values of ecological factors in countries are calculated as the mean of the values of ecological factors in stocks that these countries fish (Table SI.9).

Table SI.10. Stocks that are being fished by EU countries (2006-2010).

STOCK	*BE*	*DK*	*DE*	*EE*	*IE*	*ES*	*FR*	*LV*	*LT*	*NL*	*PL*	*PT*	*FI*	*SE*
HAKENRTN	yes	yes	yes	-	-	yes	yes	-	-	yes	-	yes	-	yes
HAKESOTH	-	-	-	-	-	yes	yes	-	-	-	-	yes	-	-
CODNEARNCW	-	yes	yes	yes	yes	yes	yes	-	-	-	yes	yes	-	-
CODNEAR	-	yes	yes	yes	yes	yes	yes	-	-	-	yes	yes	-	-
CODFAPL	-	-	yes	-	-	-	-	-	-	-	-	-	-	-
CODICE	-	-	yes	-	-	-	-	-	-	-	-	-	-	-
CODBA2532	-	yes	yes	yes	-	-	-	yes	yes	-	yes	-	yes	yes
CODKAT	-	yes	yes	-	-	-	yes	-	-	yes	-	yes	-	yes
CODIS	yes	-	-	-	yes	-	yes	-	-	-	-	-	-	-
CODVIa	-	-	yes	-	yes	-	yes	-	-	-	-	-	-	-
CODIIIaW	-	-	-	-	-	-	yes	-	-	-	-	-	-	-

Table SI.11. Ecological factors per fishing country and species.

	COD				HAKE			
COUNTRIES	ABUNDANCE	OVEREXPLOITA-TION	RECOVERY	TEMPERATURE	ABUNDANCE	OVEREXPLOITA-TION	RECOVERY	TEMPERATURE
BE	0.115	0.708	0.457	0.545	0.899	0.509	0.669	0.455
DK	0.262	0.679	0.711	0.545	0.899	0.509	0.669	0.455
DE	0.366	0.733	0.706	0.545	0.899	0.509	0.669	0.455
EE	0.336	0.679	0.772	0.545	-	-	-	-
IE	0.401	0.569	0.563	0.545	-	-	-	-
ES	0.500	0.500	0.681	0.545	0.590	0.735	0.705	0.455
FR	0.347	0.626	0.536	0.545	0.590	0.735	0.705	0.455
LV	0.009	0.857	0.953	0.545	-	-	-	-
LT	0.009	0.857	0.953	0.545	-	-	-	-
NL	0.040	-	0.528	0.545	0.899	0.509	0.669	0.455
PL	0.336	0.679	0.772	0.545	-	-	-	-
PT	0.347	0.500	0.630	0.545	0.590	0.735	0.705	0.455
FI	0.009	0.857	0.953	0.545	-	-	-	-
SE	0.024	0.857	0.740	0.545	0.899	0.509	0.669	0.455

4. Socioeconomic indicators and factors

Gear diversity (S1)

Gear Diversity was calculated considering only one indicator (SPgear) and is normalized within the sample (Table SI.12). Data was obtained from the Sea Around Us portal.

Table SI.12. Values and normalization of Gear Diversity.

Fishing Countries	Species	Gear Diversity	Normalized gear diversity	GEAR DIVERSITY
BE	Cod	6	0.238	0.238
	Hake	1	0.000	0.000
DE	Cod	9	0.381	0.381
	Hake	5	0.190	0.190
DK	Cod	8	0.333	0.333
	Hake	6	0.238	0.238
EE	Cod	8	0.333	0.333
ES	Cod	2	0.048	0.048
	Hake	15	0.667	0.667
FI	Cod	9	0.381	0.381
FR	Cod	11	0.476	0.476
	Hake	13	0.571	0.571
IE	Cod	18	0.809	0.809
LT	Cod	10	0.429	0.429
LV	Cod	10	0.429	0.429
NL	Cod	2	0.048	0.048
PL	Cod	10	0.429	0.429
PT	Cod	2	0.0476	0.0476
	Hake	7	0.286	0.286
SE	Cod	22	1.000	1.000
	Hake	13	0.571	0.571

Fleet mobility (S2)

Fleet mobility factor is composed of two indicators: $ICES_{area5}$ and $ICES_{areaEU}$. $ICES_{area5}$ refers to the mobility of countries within the EU waters, obtained by looking at the ICES areas they have caught the specific stocks in a recent 5-year period. As countries have a history of fishing in foreign areas, we also want to consider the fishing mobility prior to entering the EU in 1993. Therefore $ICES_{areaEU}$ captures whether a fishing country had higher mobility prior to entering the EU. Indices are available per country and are obtained from the historical nominal catches in the major fishing area 27 of ICES, between 1959 and 2010 (https://gis.ices.dk/geonetwork/srv/metadata/c6ab8e49-324d-4940-9e9c-0281faa65310). Indices are normalized within sample and the formula for calculating the indicator is:

$$Fleet\ mobility\ =\ \frac{ICES_{area5} + ICES_{areaEU}}{2} \qquad \text{Eq. 7}$$

Table SI13. Indicators and normalization of Fleet Mobility factor.

Countries	$ICES_{area}$	$ICES_{areaEU}$	Normalized $ICES_{area}$	Normalized $ICES_{areaEU}$	FLEET MOBILITY
BE	9.50	-2.15	0.43	0.00	0.22
DK	12.50	5.48	0.59	1.00	0.79
DE	13.22	-0.27	0.63	0.25	0.44
EE	3.60	1.66	0.12	0.50	0.31
IE	-	-	-	-	-
ES	5.27	0.37	0.21	0.33	0.27
FR	20.41	5.50	1.00	1.00	1.00
LV	4.00	2.20	0.15	0.57	0.36
LT	1.20	0.22	0.00	0.31	0.15
NL	5.16	-1.76	0.21	0.05	0.13
PL	5.22	-1.59	0.21	0.07	0.14
PT	2.88	0.61	0.09	0.36	0.22
FI	-	-	-	-	-
SE	-	-	-	-	-

Catch dependency (S3)

Catch dependency is calculated for each country and stock based on their catches between 2006 and 2010 (data obtained from their historical nominal catches in the major fishing area 27 of ICES, between 1959 and 2010, https://gis.ices.dk/geonetwork/srv/metadata/c6ab8e49-324d-4940-9e9c-0281faa65310). For each country, the dependency of a given country on each stock was calculated in relation to the catch of the species under consideration ($stockdep_{sp}$) and in relation to the total catch of the country ($stockdep_{total}$):

$$stockdep_{sp} = \frac{catch_{stock}}{catch_{sp}} \qquad \text{Eq. 8}$$

$$stockdep_{total} = \frac{catch_{stock}}{catch_{total}} \qquad \text{Eq. 9}$$

Where $catch_{stock}$ is the country catch of a given stock (annual average between 2006 and 2010), $catch_{sp}$ is the country total catch of a given species (in 2010), and $catch_{total}$ is the country's total catch of all species in 2010. Normalization is within the sample. The final formula for the Catch dependency factor is the following:

$$CATCH\ DEPENDENCY = \frac{stockdep_{sp} + stockdep_{total}}{2} \qquad \text{Eq. 10}$$

Table SI.14. Catch dependency of countries on stocks.

Country	Stock	Catch Stock 2006-2010	Catch sp 2006-2010	Total_catch	Stockdep sp	Stockdep total
Belgium	CODBA2532	0	797	16128.8	0	0
Belgium	CODFAPL	0	797	16128.8	0	0
Belgium	CODICE	0	797	16128.8	0	0
Belgium	CODIIIaW	0	797	16128.8	0	0
Belgium	CODIS	38.6	797	16128.8	0.0484	0.0024
Belgium	CODKAT	0	797	16128.8	0	0
Belgium	CODNEARNCW_CODNEAR	0	797	16128.8	0	0
Belgium	CODVIa	0	797	16128.8	0	0
Belgium	HAKENRTN	5.6	69	16128.8	0.0812	0.0003
Belgium	HAKESOTH	0	69	16128.8	0	0
Germany	CODBA2532	2577	19476	80086	0.1323	0.0322
Germany	CODFAPL	1	19476	80086	0.0001	0
Germany	CODICE	38	19476	80086	0.002	0.0005
Germany	CODIIIaW	0	19476	80086	0	0
Germany	CODIS	0	19476	80086	0	0
Germany	CODKAT	62.8	19476	80086	0.0032	0.0008
Germany	CODNEARNCW_CODNEAR	6415.8	19476	80086	0.3294	0.0801
Germany	CODVIa	1.66	19476	80086	0.0001	0
Germany	HAKENRTN	5.4	278	80086	0.0194	0.0001
Germany	HAKESOTH	0	278	80086	0	0
Denmark	CODBA2532	7625.6	26702	1066558.7	0.2856	0.0071
Denmark	CODFAPL	0	26702	1066558.7	0	0
Denmark	CODICE	0	26702	1066558.7	0	0
Denmark	CODIIIaW	0	26702	1066558.7	0	0
Denmark	CODIS	0	26702	1066558.7	0	0
Denmark	CODKAT	3014.8	26702	1066558.7	0.1129	0.0028
Denmark	CODNEARNCW_CODNEAR	1	26702	1066558.7	0	0
Denmark	CODVIa	0	26702	1066558.7	0	0
Denmark	HAKENRTN	406.8	2189	1066558.7	0.1858	0.0004
Denmark	HAKESOTH	0	2189	1066558.7	0	0
Estonia	CODBA2532	692.8	796	84965.08	0.8704	0.0082
Estonia	CODFAPL	0	796	84965.08	0	0
Estonia	CODICE	0	796	84965.08	0	0
Estonia	CODIIIaW	0	796	84965.08	0	0
Estonia	CODIS	0	796	84965.08	0	0
Estonia	CODKAT	0	796	84965.08	0	0
Estonia	CODNEARNCW_CODNEAR	15	796	84965.08	0.0188	0.0002
Estonia	CODVIa	0	796	84965.08	0	0
Spain	CODBA2532			755465.3	0	
Spain	CODFAPL	0	0	755465.3	0	0

Spain	CODICE	0	0	755465.3	0	0
Spain	CODIIIaW			755465.3	0	
Spain	CODIS	0	0	755465.3	0	0
Spain	CODKAT			755465.3	0	
Spain	CODNEARNCW_CODNEAR	7788.4	12661	755465.3	0.6151	0.0103
Spain	CODVIa	0	0	755465.3	0	0
Spain	HAKENRTN	5494.4	20681.1	755465.3	0.2657	0.0073
Spain	HAKESOTH	4695.4	20681.1	755465.3	0.227	0.0062
Finland	CODBA2532	521.7	1028	82989.1	0.5075	
Finland	CODFAPL		1028	82989.1	0	
Finland	CODICE		1028	82989.1	0	
Finland	CODIIIaW		1028	82989.1	0	
Finland	CODIS		1028	82989.1	0	
Finland	CODKAT		1028	82989.1	0	
Finland	CODNEARNCW_CODNEAR		1028	82989.1	0	
Finland	CODVIa		1028	82989.1	0	
France	CODBA2532			254876.4	0	
France	CODFAPL	0	0	254876.4	0	0
France	CODICE	0	0	254876.4	0	0
France	CODIIIaW	1.5	5298	254876.4	0.0003	0
France	CODIS	10.4	5298	254876.4	0.002	0
France	CODKAT	1	5298	254876.4	0.0002	0
France	CODNEARNCW_CODNEAR	2313.8	5298	254876.4	0.4367	0.0091
France	CODVIa	83.2	5298	254876.4	0.0157	0.0003
France	HAKENRTN	6333.8	19184	254876.4	0.3302	0.0249
France	HAKESOTH	252.6	19184	254876.4	0.0132	0.001
Ireland	CODBA2532	0	1272	247841.15	0	0
Ireland	CODFAPL	0	1272	247841.15	0	0
Ireland	CODICE	0	1272	247841.15	0	0
Ireland	CODIIIaW	0	1272	247841.15	0	0
Ireland	CODIS	422.6	1272	247841.15	0.3322	0.0017
Ireland	CODKAT	0	1272	247841.15	0	0
Ireland	CODNEARNCW_CODNEAR	172.8	1272	247841.15	0.1358	0.0007
Ireland	CODVIa	43.8	1272	247841.15	0.0344	0.0002
Lithuania	CODBA2532	237.5	3213	5535.6	0.0739	0.0429
Lithuania	CODFAPL		3213	5535.6	0	
Lithuania	CODICE		3213	5535.6	0	
Lithuania	CODIIIaW		3213	5535.6	0	
Lithuania	CODIS		3213	5535.6	0	
Lithuania	CODKAT		3213	5535.6	0	
Lithuania	CODNEARNCW_CODNEAR		3213	5535.6	0	
Lithuania	CODVIa		3213	5535.6	0	
Latvia	CODBA2532	1535.8	5160	67133.8	0.2976	0.0229
Latvia	CODFAPL		5160	67133.8	0	
Latvia	CODICE		5160	67133.8	0	
Latvia	CODIIIaW		5160	67133.8	0	
Latvia	CODIS		5160	67133.8	0	
Latvia	CODKAT		5160	67133.8	0	
Latvia	CODNEARNCW_CODNEAR	0	5160	67133.8	0	0
Latvia	CODVIa		5160	67133.8	0	
Netherlands	CODBA2532		2731	444131.5	0	
Netherlands	CODFAPL	0	2731	444131.5	0	0
Netherlands	CODICE	0	2731	444131.5	0	0
Netherlands	CODIIIaW	0	2731	444131.5	0	0
Netherlands	CODIS	0	2731	444131.5	0	0
Netherlands	CODKAT	21.2	2731	444131.5	0.0078	0
Netherlands	CODNEARNCW_CODNEAR	0	2731	444131.5	0	0
Netherlands	CODVIa	0	2731	444131.5	0	0
Poland	CODBA2532	6533.2	14841	84013.2	0.4402	0.0778
Poland	CODFAPL	0	14841	84013.2	0	0
Poland	CODICE	0	14841	84013.2	0	0
Poland	CODIIIaW		14841	84013.2	0	
Poland	CODIS		14841	84013.2	0	
Poland	CODKAT	0	14841	84013.2	0	0
Poland	CODNEARNCW_CODNEAR	1530.4	14841	84013.2	0.1031	0.0182
Poland	CODVIa	0	14841	84013.2	0	0
Portugal	CODBA2532		4724	182939.5	0	
Portugal	CODFAPL		4724	182939.5	0	
Portugal	CODICE		4724	182939.5	0	
Portugal	CODIIIaW		4724	182939.5	0	
Portugal	CODIS		4724	182939.5	0	
Portugal	CODKAT	346	4724	182939.5	0.0732	0.0019
Portugal	CODNEARNCW_CODNEAR	4379	4724	182939.5	0.927	0.0239

Portugal	CODVIa		4724	182939.5	0	
Portugal	HAKENRTN	0.5	2433	182939.5	0.0002	0
Portugal	HAKESOTH	1772.6	2433	182939.5	0.7286	
Sweden	CODBA2532	9284.6	12406	220922.7	0.7484	0.042
Sweden	CODFAPL		12406	220922.7	0	
Sweden	CODICE	0	12406	220922.7	0	0
Sweden	CODIIIaW	0	12406	220922.7	0	0
Sweden	CODIS		12406	220922.7	0	
Sweden	CODKAT	568.4	12406	220922.7	0.0458	0.0026
Sweden	CODNEARNCW_CODNEAR	0	12406	220922.7	0	0
Sweden	CODVIa	0	12406	220922.7	0	0
Sweden	HAKENRTN	56.8	71	220922.7	0.8	0.0003
Sweden	HAKESOTH		71	220922.7	0	
Finland	HAKENRTN			82989.1		
Finland	HAKESOTH			82989.1		
Finland	CODNEARNCW_CODNEAR		1028	82989.1		
Finland	CODFAPL		1028	82989.1		
Finland	CODICE		1028	82989.1		
Finland	CODBA2532	521.7	1028	82989.1		0.0063
Finland	CODKAT		1028	82989.1		
Finland	CODIS		1028	82989.1		
Finland	CODVIa		1028	82989.1		
Finland	CODIIIaW		1028	82989.1		

Note that Catch dependency in Table SI.14 is shown on a country and stock basis. In order to have the Factor per country, we averaged the catch dependency of stocks on each country.

Adaptive management (S4)

Adaptive Management has two indicators set at the country level: fisheries Management investment and fisheries Research investment. Both indicators are obtained directly from Sea Around Us (http://www.seaaroundus.org/data/#/feru). Normalization is within the sample. The formula for the factor Adaptive Management is the following:

$$ADAPTIVE\ MANAGEMENT = \frac{Research + Management}{2} \qquad \text{Eq. 11}$$

Table SI.15. Adaptive Management indicator values and normalization.

Countries	Research	Management	Normalized Research	Normalized Management	ADAPTIVE MANAGE-MENT
BE	5385	4333	0.070	0.000	0.034
DK	12472	27710	0.170	0.280	0.223
DE	8030	13325	0.110	0.110	0.106
EE	806	8862	0.000	0.050	0.029
IE	8480	5839	0.110	0.020	0.065
ES	17871	71934	0.250	0.800	0.524
FR	20341	19279	0.280	0.180	0.229
LV	617	10759	0.000	0.080	0.039
LT	528	5910	0.000	0.020	0.009
NL	12811	18128	0.170	0.160	0.169
PL	1431	88672	0.010	1.000	0.506
PT	4115	18290	0.050	0.170	0.108
FI	2295	6021	0.030	0.020	0.023
SE	71149	15606	1.000	0.130	0.567

Social factors summary

Table SI.16 shows the summary of the different social factors across stock.

Table SI.16. Summary of the social factors per stock and country.

Countries	FLEET.MOBILITY	CATCH.DEP.cod	CATCH.DEP.hake	ADAPTIVE.MNG
**BE**	0.22	0.970	0.996	0.034
**DK**	0.79	0.959	0.995	0.223
**DE**	0.44	0.764	0.999	0.106
**EE**	0.31	0.948	-	0.029
**IE**	-	0.989	-	0.065
**ES**	0.27	0.871	0.916	0.524
**FR**	1	0.977	0.838	0.229
**LV**	0.36	0.714	-	0.039
**LT**	0.15	0.464	-	0.009
**NL**	0.13	1.000	-	0.169
**PL**	0.14	0.401	-	0.506
**PT**	0.22	0.839	0.939	0.108
**FI**	-	0.921	-	0.023
**SE**	-	0.722	0.996	0.567

5. Institutional Indicators and Factors

Co-management (I1)

Co-management uses only one indicator relating the number of fishing organizations in a given country in 2017 (*N.organizations*), which is normalized within sample (Table SI.17). Data were obtained from https://ec.europa.eu/fisheries/cfp/market/producer_organisations_en.

Table SI.17. Number of organizations by country, normalization and Co-Management factor estimated.

**Country**	***N.organizations***	***N.organizations* norm.**	**Co-management**
BE	2	0.00	0.00
DK	3	0.03	0.03
DE	13	0.33	0.33
EE	7	0.15	0.15
IE	5	0.10	0.10
ES	33	1.00	1.00
FR	18	0.51	0.51
LV	4	0.08	0.08
LT	3	0.05	0.05
NL	11	0.28	0.28
PL	15	0.26	0.26
PT	16	0.36	0.36
SE	7	0.13	0.13

Property rights (I2)

Property Rights factor is composed of one indicator (Swaps), which refers to the monetary exchange of quota in a country (Andersen et al., 2009) [10]. It can be negative (the country buys quota in exchange of money) or positive (the country sells quota in exchange of money). The indicator was calculated between 2000 and 2006. Indices are normalized within the sample.

Table SI18. Indicators and normalization of Property Rights

Countries	Swaps (million € 2000 – 2006)	Swaps' normalized	PROPERTY RIGHTS
BE	8.00	0.69	0.69
DK	39.00	0.81	0.81
DE	-165.00	0.00	0.00
EE	1.00	0.66	0.66
IE	15.00	0.71	0.71
ES	87.00	1.00	1.00
FR	-49.00	0.46	0.46
LV	2.00	0.66	0.66
LT	3.00	0.67	0.67
NL	22.00	0.74	0.74
PL	8.00	0.69	0.69
PT	21.00	0.74	0.74
FI	2.00	0.66	0.66
SE	0.00	0.65	0.65

Quotas (I3)

Quotas are calculated using the Total Allowable Catch (TAC) above advice in 2015. Values were obtained from Carpenter et al. (2016) and depend on species and country, but not on stock. To calculate the percentage of TAC above advice we use the formula (TAC – ICES advice) / ICES advice * 100. Normalization is inverse within the sample.

Table SI19. Quotas factor per species.

COUNTRIES	Quotas COD	Quotas HAKE	QUOTAS COD norm (inverse)	QUOTAS HAKE norm (inverse)
BE	71.78	10.80	0.958	0.976
DK	3616.57	133.57	0.000	0.967
DE	881.78	12.93	0.739	0.976
EE	150.53	-	0.936	-
IE	222.16	-	0.917	-
ES	-84.89	1723.80	1.000	0.844
FR	429.14	608.08	0.861	0.930
LV	566.17	-	0.824	-
LT	380.53	-	0.874	-
NL	241.36	8.93	0.912	0.976
PL	1158.49	-	0.664	-
PT	-27.41	-27.41	0.984	0.979
FI	281.91	-	0.901	-
SE	1842.97	13.12	0.479	0.976

Strength (I4)

Strength is a single indicator factor built from the IUU Fishing Index [14], which measures the level of compliance with fishing regulations and effort against Illegal, Unreported, Unregulated Fishing. The compliance index ranges from 1 (best compliance) to 5 (worst compliance), and is therefore reversed in the normalization, using within sample normalization. Table SI.20 shows the original value, normalized values, and factor.

Table SI.20. Compliance indicator and Strength factor.

Countries	Compliance	Compliance' (normalized) (reversed)	STRENGTH
BE	1.430	1.000	1.000
DK	1.940	0.549	0.549
DE	1.890	0.593	0.593
EE	1.670	0.788	0.788
IE	1.850	0.628	0.628
ES	2.560	0.000	0.000
FR	2.280	0.248	0.248
LV	1.570	0.876	0.876
LT	2.020	0.478	0.478
NL	2.020	0.478	0.478
PL	1.680	0.779	0.779
PT	2.450	0.097	0.097
FI	1.670	0.788	0.788
SE	1.730	0.735	0.735

Institutional factors summary

The final set of institutional factors per species is available in Table SI.21.

Table SI.21. List of Institutional factors.

COUNTRIES	ORGANIZATION	PROPERTY.RIGHTS	STRENGTH	QUOTAS.cod	QUOTAS.hake
BE	0.00	0.69	0.600	0.958	0.976
DK	0.03	0.81	0.800	0.000	0.967
DE	0.33	0.00	0.800	0.739	0.976
EE	0.15	0.66	0.300	0.936	0.000
IE	0.10	0.71	0.000	0.917	0.000
ES	1.00	1.00	0.300	1.000	0.844
FR	0.51	0.46	0.500	0.861	0.930
LV	0.08	0.66	0.500	0.824	0.000
LT	0.05	0.67	0.400	0.874	0.000
NL	0.28	0.74	0.800	0.912	0.976
PL	0.26	0.69	0.700	0.664	0.000
PT	0.36	0.74	1.000	0.984	0.979
FI	-	0.66	0.800	0.901	0.000
SE	0.13	0.65	1.000	0.479	0.976

6. Resilience factors summary

Tables SI.22 and SI.23 show the different factors used to calculate the resilience of countries.

Table SI.22. Factors average and standard deviations.

FACTOR		mean	Standard deviation
E2	ABUNDANCE	0.47	0.18
E3	TEMPERATURE	0.36	0.21
E4	OVEREXPLOITATION	0.58	0.22
E5	RECOVERY	0.63	0.12
S1	GEAR DIVERSITY	0.50	0.51
S2	FLEET MOBILITY	0.37	0.28
S3	CATCH DEPENDENCY	0.91	0.12
S4	ADAPTIVE MANAGEMENT	0.19	0.20
I1	CO-MANAGEMENT	0.25	0.27
I2	PROPERTY RIGHTS	0.65	0.22
I3	QUOTAS	0.14	0.24
I4	DEVELOPMENT	0.60	0.35

Table SI.23. Factors average and standard deviation in index, per species.

**Factor**		**cod**		**hake**	
		mean	***sd***	***mean***	***sd***
E2	ABUNDANCE	0.41	0.19	0.60	0.05
E3	TEMPERATURE*	0.21	0.00	0.64	0.00
E4	OVEREXPLOITATION**	0.47	0.19	0.76	0.14
E5	RECOVERY	0.62	0.14	0.63	0.06
S1	CO-MANAGEMENT	0.25	0.27	0.25	0.27
S2	PROPERTY RIGHTS	0.65	0.22	0.65	0.22
S3	DEVELOPMENT	0.60	0.35	0.60	0.35
S4	QUOTAS	0.14	0.14	0.14	0.32
I1	GEAR DIVERSITY*	1.00	0.00	0.00	0.00
I2	FLEET MOBILITY	0.37	0.28	0.37	0.28
I3	CATCH DEPENDENCY**	0.88	0.14	0.98	0.03
I4	ADAPTIVE MANAGEMENT	0.19	0.20	0.19	0.20

*Factors where indicator data is not available on a country basis. **Reversed direction of factor.

7. Index performance

We compare our index to other indices in the literature that are used in climate change vulnerability and fisheries performance, following [7]. Figure SI.8 shows the results for the correlation of our resilience index with the Ocean Health Index [15], Gross Domestic Product in countries (WB), Readiness and Vulnerability [16]. COD fisheries index (ecological dimension) is positively correlated with the OHI fisheries (cod, $p<0.02$); and negatively correlated (its institutional dimension) with the Vulnerability index. For Hake, the index results are correlated with GDP (<0.01) and to the readiness index (<0.05), in both cases only the ecological resilience dimension.

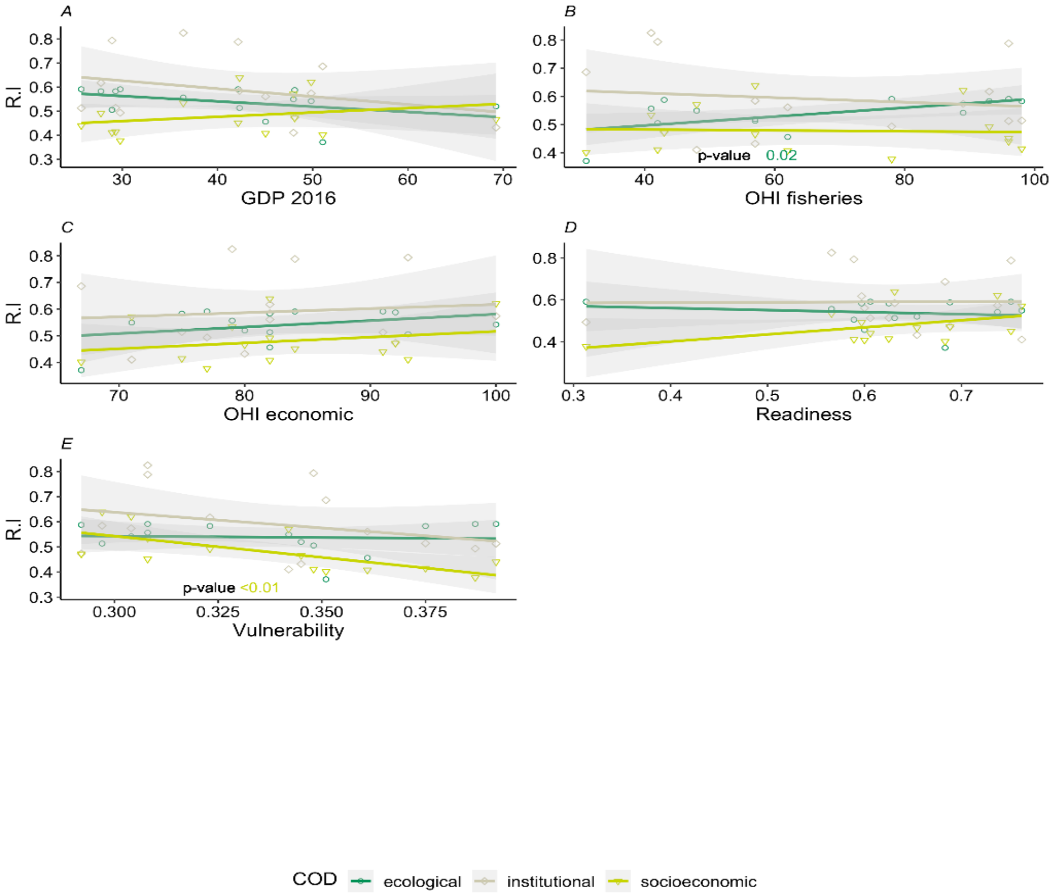

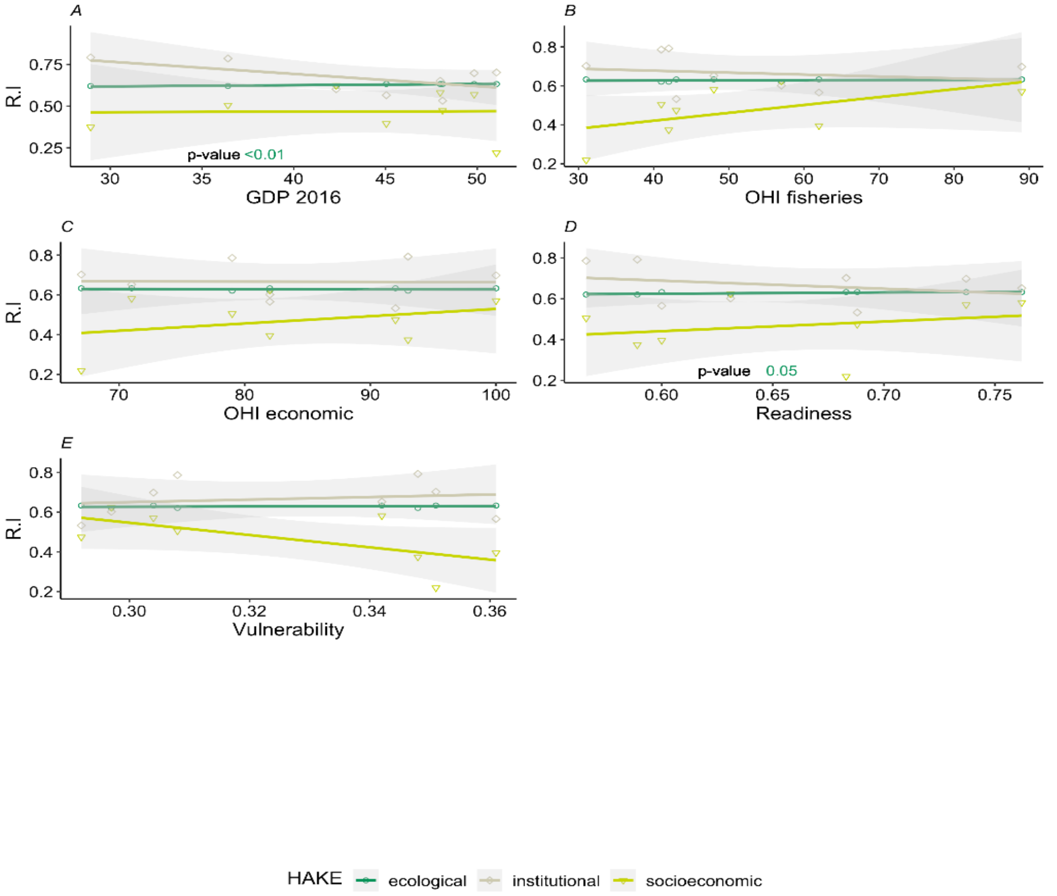

Figure SI.7. COD (above) and hake (below) resilience index comparison with country GDP, OHI, Readiness and vulnerability indices.

8. Additional analyses

We perform a Random Forest model for exploring which factors have more influence on the resilience index value. We use the “randomForest” package (version 4.6-14 [17]) in R, following Rubio et al (2020) [18] and other applications in the fisheries science (i.e., Melnychuk, Banobi, & Hilborn, 2013 [19]). Random forest models allow us to explore non-linear relationships among predictors (index factors) and the response variable (resilience index), with no assumptions made on the distribution of the response variable [18]. The random forest model we use builds 1,000 regression trees to stabilize the mean squared error (MSE) of the model. From these models the best predictors are chosen once the tree is completed, and the variable importance is shown as *%lnvMSE* (Figure 3 in main text). To explore the directionality of the predictors, we show in Figure SI.9 the partial dependence plots.

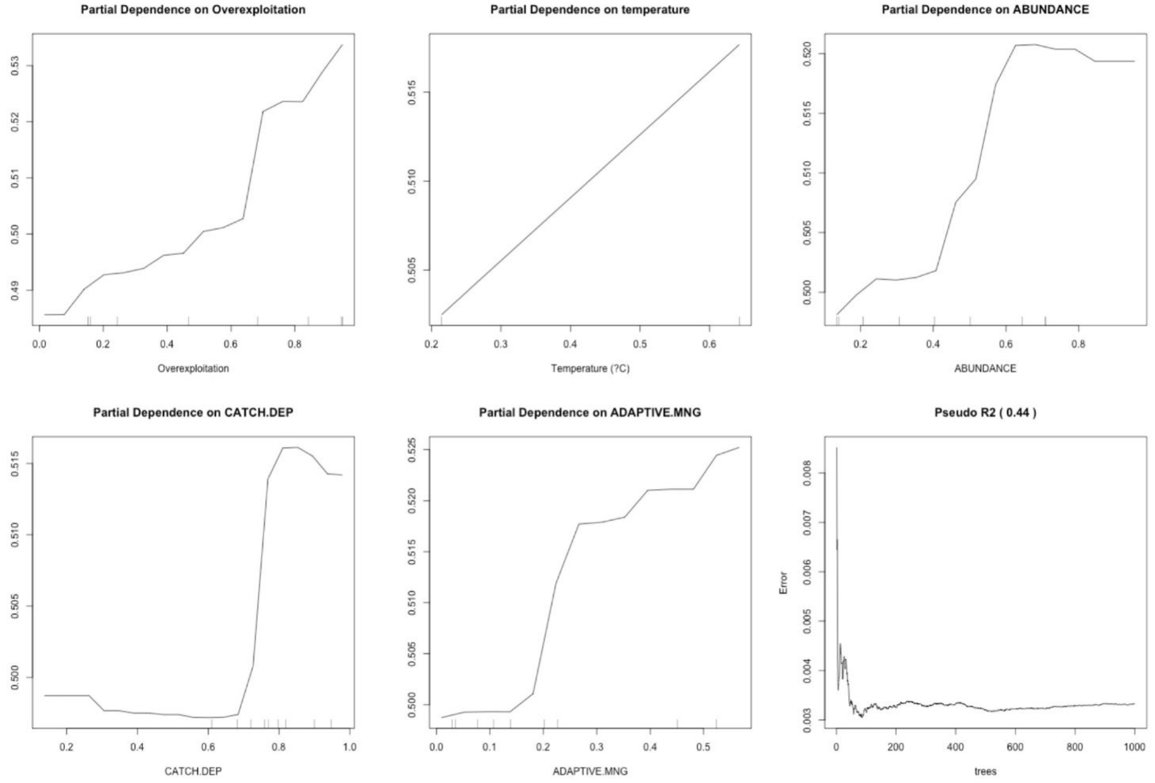

Figure SI.8. Partial dependence and Pseudo R2 of the random forest model.

We have also applied Generalized additive models (GAM) with quasibinomial family and logit link to analyze whether the resilience scores vary with latitude. GAM fitting was conducted with the ‘mgcv’ package of R [20, 21]. GAM fits for the institutional dimension of resilience as a function of latitude of countries are shown in Figure SI.10. We find differences between species, with higher institutional resilience for Hake, but no evidence of a significant effect of latitude on the index for any species.

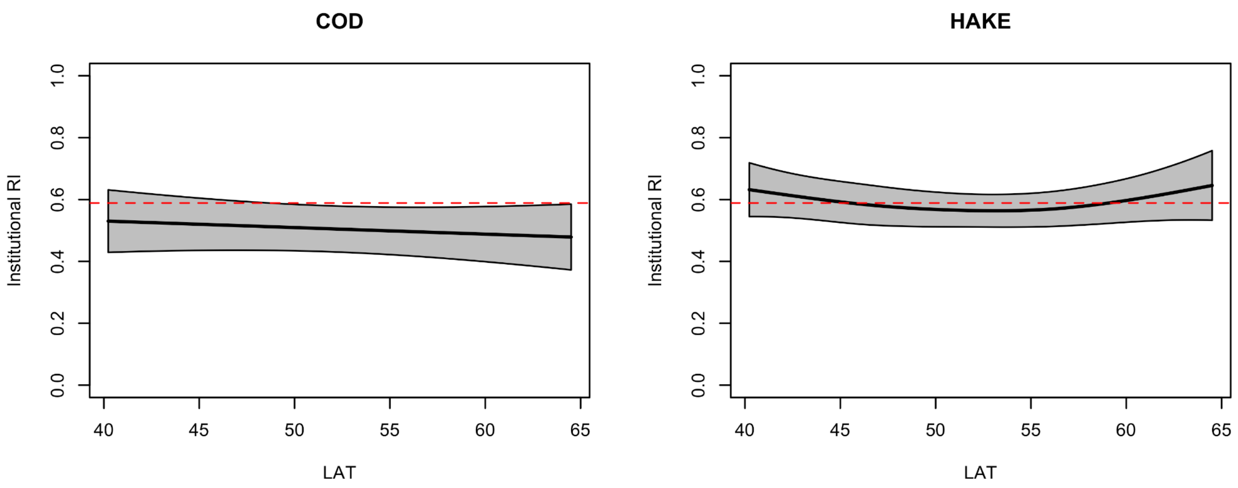

Figure SI.9 GAM fits of institutional resilience as a function of latitude by species for the institutional dimension. Estimator (black line) with 95% confidence bands (grey band), and mean institutional resilience index (dashed red line).

Finally, we count the missing indicator data across the index calculation to keep track on the uncertainty of our results. We do this as an alternative to a confidence analysis based in expert opinion on the quality of indicators, as we have not performed a stakeholder analysis and involvement in this approach. We find that missing values are very minor both for hake and cod, and the summary tables are presented in Table SI.24 and SI.25.

Table SI.24 Indicator missing values counting for the resilience index for cod.

Country	NA count-cod		
	**Ecological**	**Socio-economic**	**Institutional**
**BE**	0	0	0
**DK**	0	0	0
**DE**	0	0	1
**EE**	0	0	0
**IE**	0	2	0
**ES**	0	0	0
**FR**	0	0	0
**LV**	0	0	0
**LT**	0	0	0
**NL**	0	0	0
**PL**	0	0	0
**PT**	0	0	0
**FI**	0	1	0
**SE**	0	2	0
**Total NAs**	0	5	1

Table SI.25 Indicator missing values counting for the resilience index for hake.

Country	NA count-hake		
	**Ecological**	**Socio-economic**	**Institutional**
**BE**	0	0	0
**DK**	0	0	0
**DE**	0	0	0
**ES**	0	0	0
**FR**	0	0	0
**NL**	0	2	0
**PT**	0	2	0
**SE**	0	1	0
**Total NAs**	0	5	0

9. Bibliography

1. Ojea, E, Pearlman, I, Gaines, S. D, & Lester, S. E. (2017) Fisheries regulatory regimes and resilience to climate change. Ambio 46, 399–412.
2. Whitney, C. K, Bennett, N. J, Ban, N. C, Allison, E. H, Armitage, D. R, Blythe, J. L, Burt, J. M, Cheung, W. W. L, Finkbeiner, E. M, Kaplan-Hallam, M, Perry, I, Turner, N. J, & Yumagulova, L. (2017) Adaptive capacity: from assessment to action in coastal socialecological systems. Ecology and Society 22, 22.
3. van Putten, I. E, Jennings, S, Frusher, S, Gardner, C, Haward, M, Hobday, A. J, Nursey-Bray, M, Pecl, G, Punt, A, & Revill, H. (2013) Building blocks of economic resilience to climate change: A south east Australian fisheries example. Regional Environmental Change 13, 1313–1323.
4. Neubauer, P, Jensen, O. P, Hutchings, J. A, & Baum, J. K. (2013) Resilience and recovery of overexploited marine populations. Science (New York, N.Y.) 340, 347–9.
5. Quinlan, A. E, Berbés-Blázquez, M, Haider, L. J, Peterson, G. D, & Allen, C. (2016) Measuring and assessing resilience: broadening understanding through multiple disciplinary perspectives. Journal of Applied Ecology 53, 677–687.
6. Ricard, D, Minto, C, Jensen, O. P, & Baum, J. K. (2012) Examining the knowledge base and status of commercially exploited marine species with the RAM Legacy Stock Assessment Database. Fish and Fisheries 13, 380–398.
7. Melnychuk, M. C, Peterson, E, Elliott, M, & Hilborn, R. (2016) Fisheries management impacts on target species status. Proceedings of the National Academy of Sciences 114, 201609915.
8. Cinner, J. E, Huchery, C. Darling, E. S, Humphries, A. T, Graham, N. A. J, Hicks, C. C, Marshall, N. & McClanahan, T. R. (2013) Evaluating Social and Ecological Vulnerability of Coral Reef Fisheries to Climate Change. PLoS ONE 8, e74321.
9. Cheung et al. (2013). Signature of ocean warming in global fisheries catch. Nature 497: 365-368.
10. Andersen, J. L., Nielsen, M., & Lindebo, E. (2009). Economic gains of liberalising access to fishing quotas within the European Union. Marine Policy 33: 497-503.
11. Leslie, H. M, Basurto, X, Nenadovic, M, Sievanen, L, Cavanaugh, K. C, Cota-Nieto, J. J, Erisman, B. E, Finkbeiner, E, Hinojosa-Arango, G, Moreno-Báez, M, Nagavarapu, S, Reddy, S, Sánchez-Rodríguez, A, Siegel, K, Ulibarria-Valenzuela, J, Weaver, A, & Aburto-Oropeza, O. (2015) Operationalizing the social-ecological systems framework to assess sustainability. Proceedings of the National Academy of Sciences of the United States of America 112, 5979–5984.
12. https://ec.europa.eu/eurostat/web/main/home
13. Froese, R. & D. Pauly. Editors. 2021. FishBase. World Wide Web electronic publication. www.fishbase.org (02/2021).
14. Pauly, D, Zeller D. & Palomares M. L. D. (Editors), 2020. Sea Around Us Concepts, Design and Data (seaaroundus.org).
15. Breiman, L. (2001) Random forests. Machine Learning 45, 5-32.
16. García-Molinos et al. (2016). Climate velocity and the future global redistribution of marine biodiversity. Nature Climate Change 6: 83-88.
17. Macfadyen, G., Hosch, G., Kaysser, N. and Tagziria, L., (2019). The IUU Fishing Index, 2019. Poseidon Aquatic Resource Management Limited and the Global Initiative Against Transnational Organized Crime.
18. Halpern, B. S, Longo, C, Hardy, D, McLeod, K. L, Samhouri, J. F, Katona, S. K, Kleisner, K, Lester, S. E, O'Leary, J, Ranelletti, M, Rosenberg, A. A, Scarborough, C, Selig, E. R, Best, B. D, Brumbaugh, D. R, Chapin, F. S, Crowder, L. B, Daly, K. L, Doney, S. C, Elfes, C, Fogarty, M. J, Gaines, S. D, Jacobsen, K. I, Karrer, L. B, Leslie, H. M, Neeley, E, Pauly, D, Polasky, S, Ris, B, St Martin, K, Stone, G. S, Sumaila, U. R, & Zeller, D. (2012) An index to assess the health and benefits of the global ocean. Nature 488, 615–620.
19. Liaw, A. & Wiener, M. (2002) Classification and regression by random Forest. R News 2, 18-22.
20. Rubio, I., Ganzedo, U., Hobday, A. J. & Ojea, E. (2020) Southward re-distribution of tropical tuna fisheries activity can be explained by technological and management change. Fish and Fisheries 21, 511-521.
21. Melnychuk, M., Banobi, J. A., Hilborn, R. (2013) Effects of management tactics on meeting conservation objectives for Western North American groundfish fisheries. PLoS ONE 8, e56684.
22. R Core Team (2020). R: A language and environment for statistical computing. R Foundation for Statistical Computing, Vienna, Austria. https://www.R-project.org/
23. Wood, S. N. (2017) Generalized additive models: an introduction with R (Chapman and Hall/CRC, 2017).
